# Optimizing Self-Monitoring in a Digital Weight Loss Intervention (Spark): Protocol for a Factorial Randomized Trial

**DOI:** 10.2196/75629

**Published:** 2025-09-23

**Authors:** Michele L Patel, Abby C King, Lisa G Rosas, Gary G Bennett, Linda M Collins, John A Gallis, Amanda B Zeitlin, Priya S Talreja, Phoebe C Crosthwaite, Kayla A Collins, Annalisa W Lim, Trudy S Kim

**Affiliations:** 1 Department of Medicine, Stanford Prevention Research Center School of Medicine Stanford University Palo Alto, CA United States; 2 Department of Epidemiology and Population Health School of Medicine Stanford University Palo Alto, CA United States; 3 Department of Medicine, Division of Primary Care and Population Health School of Medicine Stanford University Palo Alto, CA United States; 4 Department of Psychology and Neuroscience Trinity College of Arts & Sciences Duke University Durham, NC United States; 5 Department of Social and Behavioral Sciences School of Global Public Health New York University New York, NY United States; 6 Duke Global Health Institute Duke University Durham, NC United States; 7 Department of Biostatistics and Bioinformatics Duke University Durham, NC United States

**Keywords:** weight loss, obesity, intervention, self-monitoring, tracking, digital health, multiphase optimization strategy, behavior change, behavioral obesity treatment, RCT, randomized controlled trial

## Abstract

**Background:**

Self-monitoring is a vital component of behavioral obesity treatment. It often involves tracking dietary intake, physical activity, and body weight. However, the optimal combination of self-monitoring strategies that maximizes weight loss is unknown. To address this gap, we leverage a framework called the multiphase optimization strategy, which facilitates the identification of an intervention’s “active ingredients” that promote weight loss and its “inactive ingredients” that have little impact, thus adding unnecessary patient effort and time demands.

**Objective:**

This study aims to examine the unique and combined weight loss effects of 3 popular self-monitoring strategies (tracking dietary intake, steps, and body weight).

**Methods:**

Spark was an optimization-randomized clinical trial that used a 2 × 2 × 2 full factorial design with 8 experimental conditions. Participants, US adults with overweight or obesity (N=176), were randomized to receive 0-3 self-monitoring strategies in a 6-month fully digital weight loss intervention. For each assigned strategy, participants were instructed to self-monitor daily via commercially available digital tools (a mobile app, wearable activity tracker, and smart scale) and received a corresponding goal (eg, a daily calorie goal) and weekly automated feedback. All participants received core intervention components, including weekly lessons and action plans informed by Social Cognitive Theory, to promote healthy eating and physical activity. Assessments occurred at baseline and at 1, 3, and 6 months. Weight was assessed objectively via a smart scale. The primary aim is to test the main effects of the 3 self-monitoring components and their interactions on weight change from baseline to 6 months. Secondary outcomes include change in BMI, caloric intake, diet quality, physical activity, and health-related quality of life, as well as 1- and 3-month weight change and the relation between self-monitoring engagement and weight change. Patterns of engagement will be operationalized as the percentage of days of self-monitoring during the 6-month intervention. Moderators of weight loss success will be explored to understand whether certain subgroups of individuals benefit more from specific self-monitoring strategies. We also conducted a separate embedded experiment to test the impact of a self-directed web-based orientation session on 6-month trial retention. After the intervention, semistructured qualitative interviews were conducted with a subset of participants to elucidate factors that impact engagement and its link to weight loss.

**Results:**

Recruitment occurred from September 2023 to November 2024. Data collection was completed in June 2025. Data analysis is ongoing.

**Conclusions:**

This trial will provide evidence as to which self-monitoring strategies are the “active ingredients” in a fully digital weight loss intervention and begin to explore which subgroups may do best with which strategies. These results have potential for public health impact by maximizing weight loss while minimizing patient burden.

**Trial Registration:**

ClinicalTrials.gov NCT05249465, https://clinicaltrials.gov/study/NCT05249465

**International Registered Report Identifier (IRRID):**

DERR1-10.2196/75629

## Introduction

### Background

With the prevalence of overweight and obesity at approximately 71% among US adults [1,2], there is a critical need for weight loss interventions that are both effective and scalable. Gold standard behavioral obesity treatments are effective in producing weight loss of 5%-8%. These 6-12 month treatments pair frequent counseling with evidence-based behavioral strategies to promote healthy dietary change and physical activity [3,4]. Despite their efficacy, these treatments are not easily scalable or accessible due to their high intensity, personnel costs, and time demands.

Digital health interventions can overcome these barriers by delivering weight loss programs remotely and in a standalone format (ie, without a human counseling component). They also have the potential to target broad segments of the population with overweight or obesity, including geographically, racially, and ethnically heterogeneous populations who are underrepresented in traditional obesity treatment research [5-8] despite being disproportionately burdened by obesity [9,10]. However, fully digital weight loss interventions produce only modest weight loss, ranging from 2% to 4% [11-19]. Thus, to fully capitalize on these highly scalable digital interventions, more work is needed to enhance their potency.

Self-monitoring is a well-established behavioral strategy for facilitating weight loss among adults with overweight or obesity [20-22]. It involves tracking a behavior (eg, dietary intake [23] and physical activity [24,25]) or a health-related outcome (eg, body weight [26]). Self-regulation theories, including the Social Cognitive Theory and the Control Theory, posit that behavior change occurs through a cyclical process of goal setting, paying attention to behaviors via self-monitoring, receiving feedback on how one’s current performance compares to one’s goal or past performance, creating an action plan to adjust behavior, and repeating the process to get closer to attaining one’s goal [27,28]. These behavioral strategies work in tandem to promote greater behavior change [29-32]. Self-monitoring is one of the strongest predictors of behavior change [29] and weight loss [31,33]. Numerous systematic reviews and meta-analyses have demonstrated that greater engagement in self-monitoring is consistently linked to greater weight loss [21-23,26,34,35]. Further, this relation holds across many intervention contexts, including human counseling–based and fully digital interventions [22]. However, self-monitoring engagement often declines over time [36-39]. Common barriers include time demands, perceived burden, accessibility challenges, waning novelty, lack of clarity on how to use such data to change behavior, and constraints related to literacy and numeracy [40-42].

One way to promote self-monitoring engagement is by using digital rather than paper-based methods of self-monitoring [22]. Advantages of using digital platforms for self-monitoring include immediate personalized feedback, time savings when using devices such as wireless activity trackers and smart scales, and high portability of mobile health tools, which increases the likelihood of engagement while reducing retrospective errors. In our systematic review of self-monitoring in digital weight loss interventions among adults with overweight or obesity, we found that 81% of interventions included self-monitoring of dietary intake, 82% included self-monitoring of physical activity, and 72% included self-monitoring of weight. In total, over half (54%) of the interventions included all 3 of these self-monitoring strategies [22]. In summary, self-monitoring is a theory-informed and evidence-based approach for weight loss that can be delivered via digital tools.

### Gap in the Field

To the best of our knowledge, no study has systematically examined the optimal combination of self-monitoring strategies included in behavioral obesity treatment. Specifically, weight loss studies often combine multiple self-monitoring domains—tracking dietary intake, physical activity, and body weight—into 1 bundled “treatment package,” which is then compared to a suitable control in an evaluation-randomized controlled trial (RCT) [21-23,34,35]. In this classical treatment package paradigm, these trials can determine the overall efficacy of a weight loss intervention but cannot disentangle the effects of individual self-monitoring components. Thus, in trials using the classical paradigm, if an intervention demonstrates superiority, it would be unclear whether all self-monitoring strategies were needed to produce clinical impact. In addition, if an intervention shows a null effect, it would be unclear whether any of the self-monitoring strategies were beneficial, or whether their effects were dampened by other burdensome intervention components. In summary, trials using the classical treatment package approach are not able to discern whether all or none of the self-monitoring strategies are valuable.

Similarly, in comparative effectiveness trial designs, the unique effects of individual self-monitoring components can be established but not their combined effects. Past studies have used these designs to isolate the effects of 1 self-monitoring domain, including self-monitoring weight (vs not) [43,44] or self-monitoring diet (vs not) [36,45,46]. Other studies have varied the content or frequency of what is being self-monitored within 1 domain (eg, comparing simplified to detailed versions of dietary self-monitoring) [47-53]. Taken together, it is unknown whether some or all 3 of these popular self-monitoring strategies are effective.

Examining whether any interaction effects exist among the 3 most common self-monitoring strategies has the potential for enhancing the efficacy of digital interventions while limiting patient burden. Interactions can be synergistic (ie, when combined, the components result in better outcomes than expected based on the main effects alone) or antagonistic (ie, worse outcomes than expected based on the main effects alone). For instance, a synergistic interaction could occur if combining self-monitoring components provided individuals with clearer insights into changing health behaviors, leading to greater weight loss than expected. In contrast, an antagonistic interaction could occur if a burdensome self-monitoring component increased the risk of disengagement from one or more other self-monitoring components, undermining the effect of those components. This antagonistic interaction results in the combined effect being smaller than what would be expected based on the main effects of the self-monitoring components.

### Leveraging the Multiphase Optimization Strategy

Intervention optimization can be used to guide the selection of self-monitoring strategies for use in weight loss interventions. To do this, the multiphase optimization strategy (MOST), an engineering-inspired framework, enables researchers to build effective multicomponent behavioral interventions through a 3-phase process of preparation, optimization of the intervention, and evaluation of the newly optimized intervention [54,55]. First, the preparation phase consists of understanding gaps in the field, selecting intervention components to test, and developing a conceptual model. Second, the optimization phase consists of conducting a fully powered optimization-RCT to rigorously test the intervention components’ unique and combined effects on the outcome of interest. Third, a subsequent evaluation phase involves testing the newly optimized intervention versus a comparator in a traditional 2-arm evaluation-RCT.

### Objective

The Spark trial, focused on the optimization phase of MOST, seeks to optimize self-monitoring in a 6-month fully digital weight loss intervention for adults with overweight or obesity. Specifically, we are examining the unique and combined effects of 3 popular self-monitoring strategies (tracking dietary intake, steps, and body weight) on 6-month weight change. In doing so, we will be able to isolate their individual effects and examine whether any synergistic or antagonistic interactions exist among them. Results will inform the development of a newly optimized intervention that retains only the self-monitoring strategies that have a clinically meaningful effect in order to maximize weight loss while minimizing patient burden. We will also assess the self-monitoring strategies’ unique and combined effects on secondary outcomes, including caloric intake, diet quality, physical activity, and health-related quality of life, as well as on 1- and 3- month weight change and the relation between self-monitoring engagement and weight change. Moderators of weight loss success will be explored to understand whether certain subgroups of individuals benefit more from specific self-monitoring strategies. Using an embedded experimental design, we will test whether a self-directed web-based orientation session—completed prior to enrollment—promotes greater 6-month retention in the Spark trial. Finally, we will conduct semistructured qualitative interviews with trial participants to explore factors that impact engagement and its link to weight loss. This paper describes the study protocol, following the SPIRIT (Standard Protocol Items: Recommendations for Interventional Trials) 2025 Checklist (see Multimedia Appendix 1), which are standardized guidelines for reporting clinical trial protocols [56].

## Methods

### Study Design and Overview

The Spark trial was an optimization RCT that tests the unique and combined effects of 3 self-monitoring strategies (tracking dietary intake, steps, and body weight) in a 6-month fully digital weight loss intervention for adults with overweight or obesity. The study used a 2^3^ full factorial design (ie, 2 × 2 × 2), whereby each participant was randomized to 1 of 8 experimental conditions that represent all possible combinations of these self-monitoring strategies. For each strategy, half of the participants were assigned to receive it (“Yes”) while the other half did not receive it (“No”; see Table 1). All participants received a core intervention that included weekly lessons and action plans for promoting healthy eating and physical activity. We recruited 176 participants (22 per condition). The trial was preregistered in February 2022 at ClinicalTrials.gov: NCT05249465. The trial registry includes the study protocol [57]. No patient involvement occurred, though study procedures and intervention content were adapted from the principal investigator’s (PI’s) prior digital weight loss trials (ie, GoalTracker [36], Spark Pilot [47], and Ignite Pilot [58]) that gathered qualitative participant feedback on perceived satisfaction and difficulty of intervention components, feasibility of study procedures, and suggestions for improvement.

**Table 1:** Factorial design (2 × 2 × 2) testing three self-monitoring strategies in the Spark trial.

Experimental condition	Core	Track diet	Track steps	Track weight
1	Yes	No	No	No
2	Yes	No	No	Yes
3	Yes	No	Yes	No
4	Yes	No	Yes	Yes
5	Yes	Yes	No	No
6	Yes	Yes	No	Yes
7	Yes	Yes	Yes	No
8	Yes	Yes	Yes	Yes

### Participants

The inclusion and exclusion criteria are listed in Textbox 1.

**Inclusion and exclusion criteria.**

**Inclusion criteria**

- Adults aged ≥18 years.
- BMI of 25.0-45.0 kg/m^2^, which corresponds to having overweight or obesity [59].
- Smartphone ownership and access to a personal email account.
- Willing to install the Fitbit mobile app (Google) on one’s personal phone.
- Proficiency in the English language.
- Living in the United States.
- Interested in losing weight through behavioral strategies.

**Exclusion criteria**

- Concurrent enrollment in another weight management intervention.
- Loss of ≥10 lbs (ie, 4.5 kg) in the past 6 months.
- Current use of a weight loss or antiobesity medication.
- Prior or planned bariatric surgery.
- Current, recent, or planned pregnancy during the study period.
- Currently breastfeeding or lactating.
- Living with someone else participating in the study.
- Inability to engage in moderate forms of physical activity akin to brisk walking (assessed via the Physical Activity Readiness Questionnaire for Everyone [PAR-Q+] [60])
- Potential contraindications to losing weight due to a serious medical condition (eg, cancer or dementia) or medication (eg, steroids).
- A history of an eating disorder or cardiovascular event or uncontrolled diabetes mellitus or uncontrolled hypertension that could predispose an individual to be better suited for a more intensive or different type of intervention.
- Investigator discretion for safety reasons.

### Study Procedures

**Figure 1:**
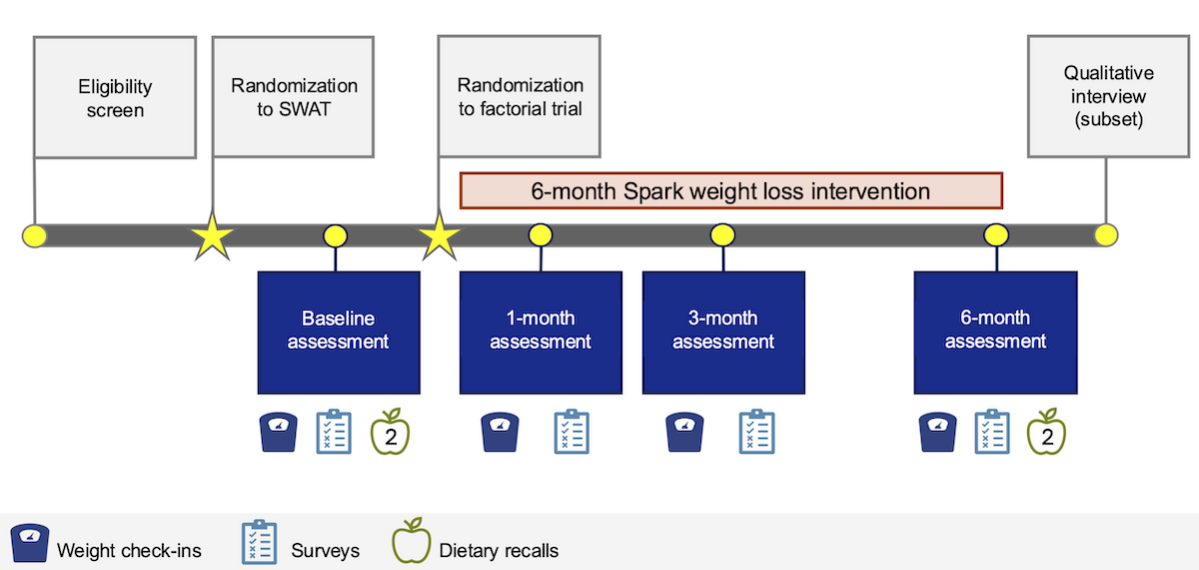
Study procedures diagram in the Spark trial. SWAT: study within a trial.

The study procedures are depicted in Figure 1.

#### Recruitment

The study was fully remote and was delivered across the United States. Enrollment occurred on a rolling basis until our target sample size was met. We aimed for at least 50% recruitment of US racial or ethnic minority group members, given their underrepresentation in behavioral weight loss trials [6,7] and the disproportionate burden of obesity among Black and Hispanic adults in the United States [61]. We recruited participants via remote channels, including ResearchMatch (a web-based US national registry of volunteers interested in participating in health-related studies), an institute-specific diabetes registry, and ClinicalTrials.gov (a globally recognized registry of clinical trials). Recruitment materials included a brief description of the study and eligibility criteria, along with a link to a web-based eligibility screen on REDCap (Research Electronic Data Capture; Vanderbilt University), a secure, web-based software platform hosted at Stanford University. [62] Those who were eligible were prompted to provide contact information at the end of the screen.

#### Self-Directed Orientation Session

Eligible individuals were randomized 1:1 to either receive a self-directed orientation session or not receive it as part of an embedded experiment that began prior to the Spark weight loss trial (see preregistration repository: protocol SWAT (Study Within A Trial) 211 [63]). Participants in the SWAT control arm were sent an automated email prompting them to sign up for a baseline visit using a scheduling website (Calendly). Those in the SWAT intervention arm were sent an automated email prompting them to complete the self-directed orientation session, lasting approximately 20-25 minutes. This web-based orientation session consisted of video content and interactive activities (using Articulate [Articulate Global LLC] e-learning software) geared at explaining the rationale for the study and for randomization, setting clear expectations of study activities, visualizing the time commitment via a graphic timeline, and ensuring the trial was a good fit for their interests by self-generating pros and cons for joining the trial. The orientation session was modeled after the innovative Methods—Motivational Interviewing Approach [64,65], which was developed to increase retention in clinical trials by empowering potential participants in making an informed decision about trial participation prior to enrollment. At the end of the orientation, individuals had the option to sign up for a baseline visit using the scheduling website. Thus, it was possible for some participants to enroll in the embedded experiment without enrolling in the weight loss trial. These types of embedded experiments are often referred to as a Study-Within-A-Trial (ie, “SWAT”) and facilitate empirically testing strategies to promote recruitment or retention in clinical trials [66,67].

#### Baseline Tasks

All participants attended a remote baseline visit with trained study staff held via Zoom (Zoom Communications, Inc) videoconference. This visit lasted approximately 1.5 hours in length and focused on reviewing the purpose and procedures of the study, confirming eligibility, obtaining informed consent using REDCap’s electronic signature feature, installing the free, commercially available Fitbit mobile app, completing a dietary recall assessment, and administering a health literacy assessment. The study staff created a unique Fitbit account for each participant and ensured that participants could log into it. At the end of the baseline visit, the participants received a link to the baseline survey. Once completed, study staff mailed an smart scale (Fitbit Aria Air) to the participant’s home. Once the scale was delivered, participants received an email that provided information on syncing their device with the Fitbit app and prompted them to weigh themselves the following morning using a standardized protocol (see “Data collection and measures” section, below). Study staff were available for troubleshooting via phone or email to assist in syncing devices.

#### Randomization to Factorial Trial

Following recommendations for factorial designs [68], we randomized participants to the 8 conditions using restricted randomization stratified by sex assigned at birth and using permuted block randomization with a block size of 8. The allocation sequence was generated using SAS (SAS Institute Inc) by the study’s statistician (JAG) and stored in REDCap [69]. Study staff used REDCap to implement the random allocation sequence (ie, randomize a participant) once both the baseline weight and web-based surveys were submitted. If a participant was randomized to a condition that tracks steps (ie, conditions 3, 4, 7, and 8), then study staff mailed them a Fitbit activity tracker (Inspire 3) within 2 business days. Once delivered, instructions on syncing the tracker with the Fitbit app were emailed. The first day of the weight loss intervention occurred approximately 1 day after the tracker arrived (if in conditions 3, 4, 7, or 8) or 4 days after randomization (if in conditions 1, 2, 5, or 6). We chose 4 days based on the mean number of days for the tracker to be delivered once ordered. This period enabled standardization of the number of days between randomization and the start date across all conditions. On the first day of the weight loss program, participants received an automated email describing their treatment assignment (see “Intervention” section, below).

#### Masking

Participants were not masked to treatment assignment, by design, as they were informed before enrollment about the 3 types of self-monitoring to which they could be assigned. Study staff were not masked to treatment assignment due to logistical limitations and the need for quality assurance. However, all surveys were sent via REDCap and were completed without study staff involvement. In addition, study staff did not have access to the allocation sequence. The study statistician (JAG) will analyze the primary outcome in a masked fashion such that treatment assignment will not be revealed.

#### Quality Assurance

During their baseline visit, participants were asked to refrain from self-monitoring behaviors that were not assigned to them. The study’s research question and expectations were explained prior to enrollment to provide an opportunity for potential participants to decide whether the study would be a good fit. We have found high adherence to these requests in our prior trial [36]. In the first couple of weeks of the intervention, study staff verified whether participants were self-monitoring correctly (ie, tracking what they were asked to track and not tracking what they were asked not to track) through review of objective self-monitoring data. If a deviation occurred, the staff member flagged it, and the participant was sent a one-time email via REDCap reminding them of their self-monitoring assignments.

### Intervention

#### Overview

**Figure 2:**
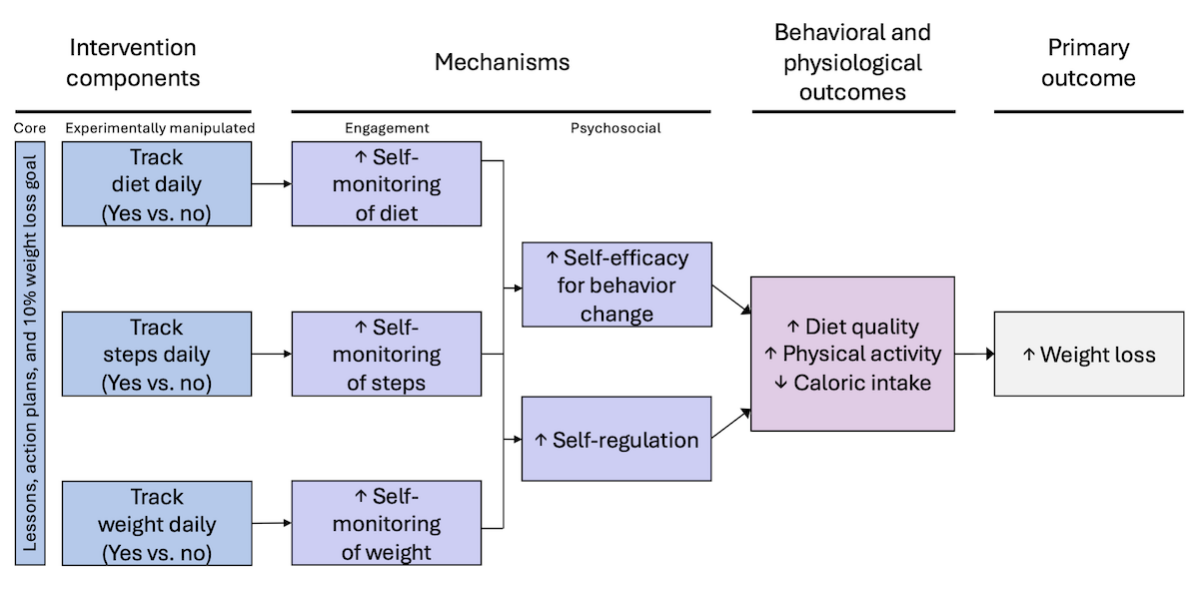
Conceptual model of the weight loss intervention in the Spark trial.

All participants received a 6-month behavioral weight loss intervention, which was empirically based [29-31,33,70-75]. This intervention was fully digital, meaning that no human counseling was provided, which was designed to promote scalability and broader reach. All participants received core components of an overall 10% weight loss goal and weekly behavioral lessons and action plans. Our conceptual model (Figure 2) outlines how we envision the self-monitoring strategies promoting weight loss. Each strategy being tested has an expected mechanism of action (ie, increased engagement in self-monitoring), which, in turn, is expected to enhance self-efficacy and self-regulatory skills, as posited by the Social Cognitive Theory and the Control Theory [27,28]. Together, these psychosocial and engagement mechanisms are expected to promote behavior change (ie, improvement in diet and physical activity), which is anticipated to create a caloric deficit, leading to weight loss. A summary of intervention components is provided in Table 2.

**Table 2:** Intervention components and digital modalities in the Spark trial.

Intervention component			Digital modality		Experimental components						
					Core		Track diet		Track steps		Track weight
**Daily self-monitoring**											
	Dietary intake	Fitbit app				✓^a^					
	Steps	Activity tracker (Fitbit Inspire 3)						✓			
	Body weight	Smart scale (Fitbit Aria Air)								✓	
**Goals^b^ and weekly tailored feedback^c^**											
	Calories	Emailed progress report				✓					
	Steps	Emailed progress report						✓			
	Weight loss, weekly	Emailed progress report								✓	
	Weight loss, overall	Emailed progress report		✓							
**Weekly skills training**											
	Lessons	Emailed PDF handout		✓							
	Action plans	Emailed link to Qualtrics survey		✓							

^a^Check mark indicates that the component was received by participants receiving the experimental condition.

^b^On the first day of the intervention, participants were emailed a Goal Sheet.

^c^Every day, participants could also view their progress via the Fitbit app.

#### Core Components

All participants received a goal of 10% weight loss by 6 months. This goal is consistent with obesity treatment guidelines [3]. Each week, participants received evidence-based skills training materials, including lessons and action plans. These were sent via email and were adapted from gold standard weight loss curriculum [70]. The lessons ere electronic handouts focused on nutrition, physical activity, and behavior change topics (see Table 3). Each lesson had a corresponding action plan (administered via a Qualtrics [Qualtrics, LLC] survey) that incorporated motivational interviewing [76,77] and problem-solving strategies [78]. Specifically, participants were guided through a series of questions to reflect on their current behaviors and areas for change, generate actionable steps related to the week’s lesson, reflect on their confidence level in doing so, and brainstorm potential barriers and solutions. Reminders were sent 4 days after the initial email to participants who had not yet completed that week’s action plan.

**Table 3:** Topics of 19 lessons in the Spark trial.

Lesson number	Week	Topic
1	1	Overview of the Spark weight loss program
2	2	Physical activity: the basics
3	3	Increasing Green Zone Foods
4	4	Navigating the grocery store
5	5	Reading food labels
6	6	Decreasing Red Zone Foods
7	7	Paying attention to portion control
8	8	Reducing added sugar
9	9	Understanding obesity
10	10	Preparing meals at home
11	11	Stepping up to physical activity
12	12	Managing emotional eating
13	14	Planning ahead for eating out
14	16	Staying motivated
15	18	Being aware of environmental cues
16	20	Identifying social cues and social support
17	22	Planning for special occasions
18	24	Taking charge of thoughts
19	26	Maintaining progress

#### Intervention Components Being Experimentally Manipulated

##### Overview

Participants received 0-3 possible self-monitoring strategies (dietary intake, steps, and body weight; see Table 1). Each strategy involved daily self-monitoring instructions, a personalized goal [29,30], and tailored feedback [32,79-81]. These 3 self-regulation strategies work in tandem to promote behavior change [29-31]. Feedback was provided each week via a “progress report” in the format of an electronic handout sent via email. It captured progress on goals (described below) as well as on the number of days of self-monitoring each week. A reminder of goals was provided in each progress report. Participants could also view real-time, graphical feedback in the Fitbit app. The app was available on both iPhone and Android platforms and was set up by study staff after randomization (prior to the first day of the intervention) to reflect the participant’s self-monitoring domains (dietary intake, steps, body weight) and corresponding goals (calories, steps, weight loss).

##### Component 1: Self-Monitoring of Dietary Intake

Participants randomized to receive this component were instructed to self-monitor their dietary intake daily via the Fitbit app. The app allowed users to track all foods and beverages consumed using a built-in nutritional database, barcode scanner, or manual entry. To aid in ease of use, recently and frequently eaten items were quickly accessible. The app automatically summed daily caloric intake and allowed users to view graphs of caloric intake over time. Participants assigned to dietary self-monitoring received a daily calorie goal based on their anticipated weekly rate of weight loss [82], calculated using data reported at baseline, including age, sex, weight, and height. For safety, the minimum daily calorie goals that could be assigned were 1200 kcal for women and 1500 kcal for men, based on national guidelines [59].

##### Component 2: Self-Monitoring of Steps

Participants randomized to receive this component were instructed to self-monitor their step count daily via a wrist-worn activity tracker (the Fitbit Inspire 3). In conjunction with the self-monitoring goal, a daily step count goal was provided that adapted based on weekly progress. The initial week’s step goal was based on the participant’s baseline scores on the Godin Leisure-Time Exercise Questionnaire (GLTEQ) leisure score index [83,84], with scores ranging from 0 to 13 (interpreted as “insufficiently active”) assigned to a goal of 5000 steps per day, scores of 14-23 (“moderately active”) assigned to 7000 steps per day, and scores ≥ 24 (“active”) assigned to 10,000 steps per day. Starting in the second week of the intervention, an adaptive step goal was given using an empirically tested algorithm [85,86] that was previously adapted and tested in our team’s prior pilot study [47]. The algorithm calculated the 60th percentile of the past week’s daily step counts, rounded up to the nearest multiple of 50, and assigned that as the subsequent week’s daily step goal. For example, a week with daily steps of 5000, 5100, 6000, 6500, 7000, 8200, and 8500 (ranked from lowest to highest) would result in a daily step goal of 6800 for the subsequent week. The new step goal appeared in each week’s progress report. The Fitbit activity tracker was synced with the Fitbit app to allow participants to view their progress toward the step goal.

##### Component 3: Self-Monitoring of Weight

Participants randomized to receive this component were instructed to self-monitor their body weight daily via a Bluetooth-enabled smart scale (the Fitbit Aria Air scale). The smart scale was synced with the Fitbit app, providing graphical feedback on weight change. These participants received a weekly weight loss goal of 0.5-2.0 lbs (0.23-0.91 kg) per week, depending on how much weight loss was required at baseline to achieve 10% weight loss at 6 months based on their baseline weight. This goal remained the same throughout the 6-month intervention. It appeared in their Fitbit app as well as in each week’s progress report.

#### Data Collection and Measures

**Figure 3:**
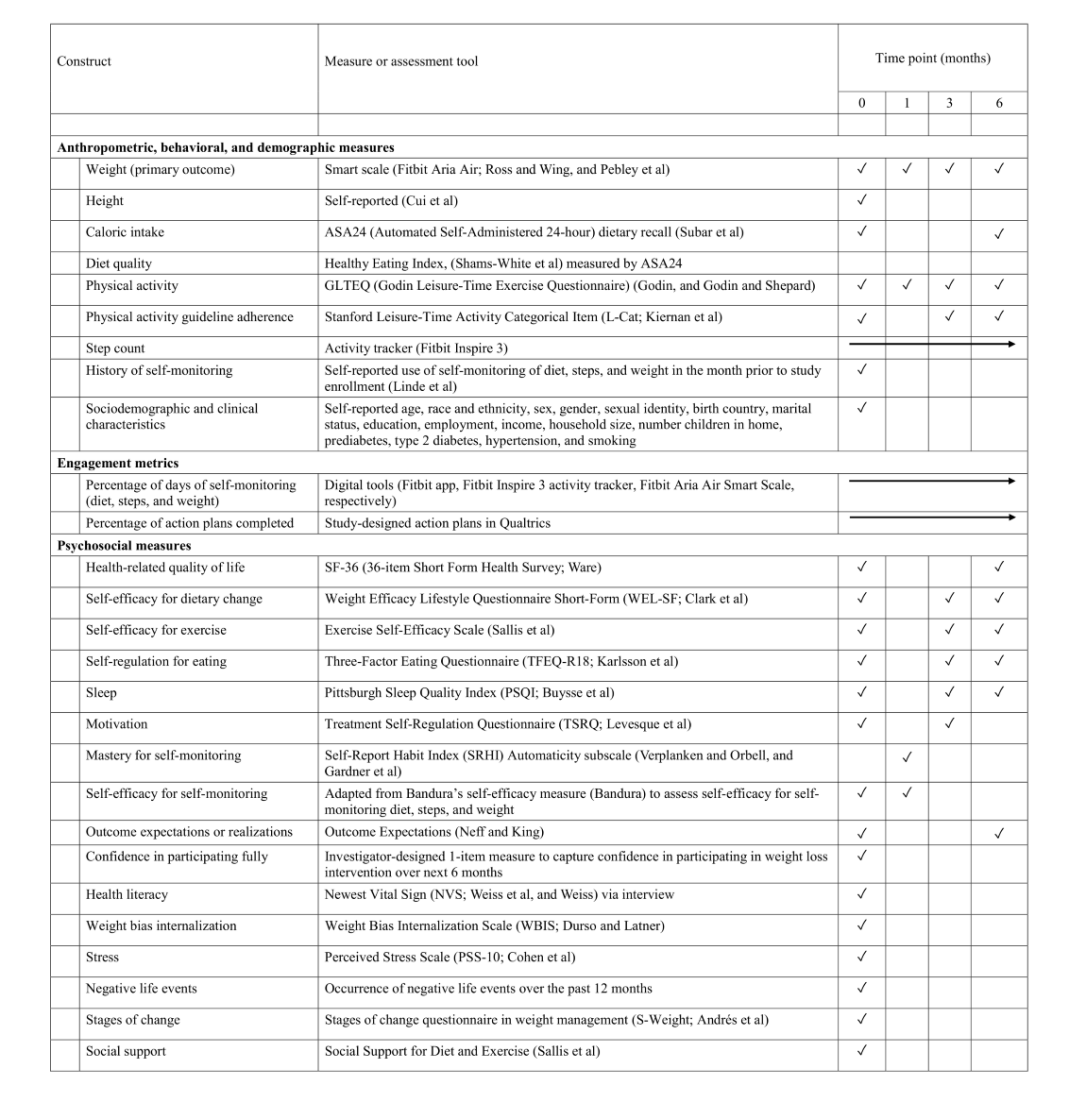
Assessment schedule in the Spark trial.

Study assessments were conducted remotely at all 4 time points: baseline, 1 month, 3 months, and 6 months (see Figure 3 [83,84,87-109]). At each assessment, participants received an automated email instructing them to weigh themselves on the smart scale, input values into a web-based weight check-in form, and complete a web-based survey. REDCap was used for survey administration and data management. Reminders to complete these tasks were automatically sent via REDCap. If the surveys were not completed after several days, study staff reached out to participants via text message, email, or phone call. Confidentiality of participant data was maintained by storing data in secure electronic files accessible only to trained study team members, prioritizing using participant ID numbers instead of names, and presenting study data in aggregate.

#### Primary Outcome Measure

Consistent with our trial preregistration (NCT05249465), our primary outcome was weight change from baseline to 6 months. Negative values indicate weight loss, whereas positive values indicate weight gain. Weight was obtained using a commercially available smart scale (Fitbit Aria Air).

Participants were asked to follow standardized procedures [110]:

1. Place the scale on a hard, flat surface.
2. Remove all articles of clothing and accessories.
3. Weigh on the scale in the morning before eating or drinking and after emptying their bladder.
4. Step on the scale and record the value.
5. Repeat it 2 more times for a total of 3 weight measurements per time point.

Weights synced from the smart scale to the Fitbit app via Bluetooth. As a preventive measure in case of syncing errors, participants were also asked to input their weight values on a web-based weight check-in form. There is high concordance between weights measured from commercial smart scales and those from scales used in a clinical setting [87,88].

#### Secondary Outcome Measures

Weight was also assessed at 1 and 3 months via the same protocol described above. BMI will be computed as the weight in kilograms divided by the height in meters squared, and the change in BMI from baseline at 1, 3, and 6 months will be reported.

Caloric intake was assessed via the Automated Self-Administered 24-hour (ASA24) Dietary Assessment Tool (versions 2022 and 2024), which is a free web-based tool developed by the National Cancer Institute [90]. Participants were asked to complete a total of 4 24-hour dietary recalls, including 2 at baseline and 2 at 6 months. At both time points, we requested 1 weekday recall and 1 weekend day recall. We sent up to 4 reminders via email or SMS text message per time point. We will exclude from our analyses any recalls with outliers of daily caloric intake reported as <600 kcal or >4400 kcal for women and <650 kcal or >5700 kcal for men, in accordance with ASA24 recommended procedures based on NHANES (National Health and Nutrition Examination Survey) data [111]. To compute caloric intake at each time point, we will calculate the mean of the weekday recall and the weekend-day recall. If only one recall is available at a given time point, we will use that value. Diet quality will be assessed using the Healthy-Eating Index-2020 [91], calculated from ASA24 data.

Physical activity was assessed via the GLTEQ, a self-report measure that assesses the past week’s frequency of different types of exercise (strenuous, moderate, and mild or light) that were engaged in for more than 15 minutes during one’s free time [83,84]. Strenuous activities were described as those where one’s “heart beats rapidly” (eg, running, jogging, or swimming), moderate activities were described as “not exhausting” (eg, fast walking or tennis), and mild activities were described as requiring “minimal effort” (eg, yoga or easy walking). A leisure score index will be created using the following formula: (strenuous × 9) + (moderate × 5) + (light × 3), with higher scores indicating more frequent exercise. To assess weekly moderate-to-vigorous physical activity, a composite score will be created using the same procedures but excluding the light activities. From this moderate-to-vigorous physical activity score index, scores of ≥24 units will be interpreted as “active,” and scores <24 will be considered “insufficiently active” [112].

We also objectively assessed step count using the Fitbit Inspire 3 activity tracker for those participants assigned to track steps (conditions 3, 4, 7, and 8). In our analyses, we will operationalize the week 1 step count as the average of the first 7 days of the intervention and the 6-month step count as the average of the last 7 days (week 26), so long as ≥3 valid days per week are reported [113]. Fitbit activity trackers have shown acceptable to excellent validity for step measurements [114].

Health-related quality of life was assessed using the 36-item Short Form Health Survey (SF-36) [94], with scores provided for both the physical and mental components. It captures 8 domains: physical functioning, role-physical, bodily pain, general health, vitality, social functioning, role-emotional, and mental health.

Self-monitoring engagement was assessed objectively for all 182 days (6 months) of the intervention using data collected from the Fitbit digital tools. Data were pulled from the Fitbit app and retrieved via Fitabase (Small Steps Lab, LLC), a software data management platform. In our analyses, engagement will be operationalized as the percentage of days in the intervention that participants self-monitored dietary intake, steps, and weight (reported separately for each domain). For self-monitoring of diet, we will count days as valid if they have ≥800 kcal recorded, which is a threshold that best predicted weight loss [37] and is commonly used for determining minimum adherence [36,115,116]. For self-monitoring of steps, we will count days as valid only if ≥1000 steps are recorded. This minimum threshold is used to minimize the possibility of inaccurately counting days with high amounts of nonwear time [117]. We also assessed engagement in other intervention components, including the percentage of action plans completed, which was objectively assessed via Qualtrics, with 100% indicating completion of all 19 action plans. Using self-report at 6 months, we assessed which of the 19 lessons were read and the frequency with which progress reports were reviewed, with options of “weekly,” “less than 1 time per week,” “less than 1 time per month,” and “never.”

Putative moderators were assessed via self-report survey measures administered at baseline, including demographic (eg, sex, age, and education), psychosocial (eg, stress and self-efficacy), behavioral (eg, pretreatment caloric intake and physical activity), and clinical (eg, diabetes diagnosis) characteristics. A health literacy questionnaire, the Newest Vital Sign, was administered orally by study staff at the baseline visit [104]. In exploratory aims, change in psychosocial outcomes were assessed via self-report survey measures over time. At the 1-month assessment, 2 measures (self-efficacy for self-monitoring and mastery for self-monitoring) specifically asked about only the domains that a given participant was instructed to self-monitor (ie, diet, steps, and weight).

Adverse events were documented via a REDCap report and reviewed by our Independent Safety Officer. If serious adverse events occurred, they were reported promptly to the Institutional Review Board and the National Institutes of Health.

Acceptability data were collected via survey to assess perceived helpfulness and perceived difficulty of intervention components.

#### Outcomes of the Embedded Experiment

Our embedded experiment tests whether implementing a self-directed web-based orientation session improves the Spark trial’s 6-month retention rate (the primary outcome of embedded experiment). Retention is operationalized as the percentage of participants who report weight at 6 months out of total participants in the Spark trial (N=176). We will also examine the orientation session’s impact on the secondary outcomes listed in Textbox 2.

**Secondary outcomes in the embedded experiment**

- 1- and 3-month retention in the Spark trial.
- Weight change from baseline to 6 months.
- Proportion of participants achieving ≥5% weight loss at 6 months from baseline.
- Engagement in self-monitoring over the 6-month intervention.
- Proportion of the 391 participants in the embedded experiment who enrolled in the Spark trial.
- Whether orientation sessions affect characteristics of the sample who enroll in Spark.
- Perceived value of retention.
- Knowledge of the importance of retention.
- Completion rates of the orientation session.

### Qualitative Interviews

After the end of the intervention, a subset of participants were invited to participate in a one-to-one semistructured qualitative interview, held via Zoom videoconference. Interviews lasted 30-45 minutes. Using an interview guide, trained research team members asked participants about factors that impacted self-monitoring engagement, perceived helpfulness, and satisfaction of the intervention components (eg, goals, self-monitoring, lessons, action plans, and progress reports), contributors and barriers to weight loss, perceived changes in self-efficacy, and to what extent self-monitoring contributed to weight loss. To contextualize and tailor their questions, interviewers integrated each participant’s engagement and weight loss outcomes as well as their 6-month acceptability data. Example questions included “You tracked your food [*x%*] of days over the 6-month Spark program. What factors contributed to tracking that amount?” and “You rated tracking your food as [“not at all” up to “very”] helpful. What contributed to feeling this way?”

Interviews were recorded and then transcribed verbatim using a professional transcription service. Memos were used by interviewers to reflect and record emerging themes. Data saturation was achieved once no new themes emerged from the interviews, at which point we stopped recruitment for the interviews. Transcripts will be coded using an iterative team-based approach to reduce potential bias. A cloud-based software platform (Dedoose; SocioCultural Research Consultants, LLC) will be used to facilitate the coding process. A codebook was created both deductively (derived from the PI’s experience and from the research questions) and inductively (derived from an initial set of transcripts). Data will be analyzed using thematic analysis. A mixed methods approach will be used to integrate the data [118,119]. providing insight into how self-monitoring engagement and weight loss are or are not related, and if so, why (ie, mechanisms). Joint display tables will be used to depict how the qualitative data provide context to the quantitative outcomes from the trial [120]. The design and reporting of the qualitative study will adhere to the COREQ (Consolidated Criteria for Reporting Qualitative Research) checklist [121].

### Statistical Analysis

Unlike an RCT that compares the means of each arm to one another (1 vs 2 vs 3, and so on), in a factorial trial, the means across combinations of conditions are used to test main effects of components and their interactions [122]. In our 2^3^ full factorial trial (ie, a 2 × 2 × 2 with 8 conditions), we are testing whether 3 self-monitoring strategies, alone or in combination, improve weight loss at 6 months (our primary time point). To estimate the main effect of each self-monitoring component on weight change, we will examine the difference between the mean of conditions where the target component is present and the mean of conditions where the target component is absent [54]. For example, for “Tracking Diet,” the mean of experimental conditions 5, 6, 7, and 8 (ie, the component is present) will be compared to the mean of conditions 1, 2, 3, and 4 (ie, the component is absent; see Table 1). We will use effect coding (ie, –1 when a component is absent and 1 when a component is present) for analyzing the main effects and interactions of the components, as recommended for factorial designs [54]. Thus, a main effect is interpreted as the effect of a factor averaged across all other factors. Since each main effect and interaction is based on the entire sample, the factorial design is considered efficient and economical [123].

### Sample Size and Statistical Power

Using a power calculation, we determined that a sample size of 176 participants would be required in our factorial trial to detect a minimum clinically important difference of 2.4 kg at 6 months, which is associated with improvements in cardiometabolic risk factors [59]. We assumed an SD of 4.5 kg, based on prior 6-month weight loss outcomes in digital interventions [49,124,125]. Thus, we sought to detect a main effect or interaction effect of Cohen *d*=0.53. Specifically, assuming 80% power, an *α* of 5% in a 2-sided test, and 35% attrition at 6 months (a conservative estimate based on prior trials of fully digital weight loss interventions [22,36,50,126]), a sample size of 172 is needed. To obtain an equal number of participants in each treatment condition, we increased the sample size to 176 total participants (22 per condition). Allowing for an equal number of participants per condition permits the tests of the main effects and interactions to be uncorrelated. Even if unequal sample size across conditions occurs due to differential dropout resulting in minor imbalance, the correlations between effects should be small [122]. This power analysis was computed using the FactorialPowerPlan SAS Macro (version 1.0) [127].

Our trial uses a 2^3^ factorial design since it has 3 factors, each with 2 levels (Yes and No). In these types of 2*^k^* factorial trial—those with *k* number of factors—all else being equal, the power for detecting a β-weight corresponding to an interaction effect is the same as that for detecting a β-weight corresponding to a main effect when using effect coding to represent the levels of the factors [54]. Thus, no additional participants are needed to detect interactions in 2*^k^* factorial trials.

### Analytic Plan for Primary Outcome

Our primary outcome is weight change, in kilograms, from baseline to 6 months. Using intent-to-treat principles, we will use linear mixed models to examine the main effects of the 3 self-monitoring components and all of their interactions on mean weight change at each follow-up time point. This model will include fixed effects for the following: time point (1, 3, or 6 months), sex assigned at birth (ie, the stratification variable), self-monitoring component, pairwise interactions of each component, and the time-by-component and time-by-pairwise interactions. We will fit an unstructured covariance matrix to take into account the correlation of weight measures within a person across time. We will use maximum likelihood to estimate the model parameters, allowing for missingness at random. Data will also be disaggregated by sex in exploratory analyses.

### Analytic Plan for Other Outcomes

#### Secondary Outcomes

Similar linear mixed models will be used to assess the main effects and interactions on the secondary outcomes, including BMI, caloric intake, diet quality, physical activity, and health-related quality of life (see Figure 3). To assess differences in achieving clinically significant weight loss (≥5% from baseline) [59] between the “present” versus “absent” levels of each factor, we will fit a log-Poisson GEE (generalized estimating equations) model [128] with robust SEs [129,130] and an unstructured working correlation matrix to take into account repeated measures across time. From these models, we will report risk ratios comparing levels of intervention components and their interactions. As recommended by the CONSORT (Consolidated Standards Of Reporting Trials) statement for randomized trials [131], we will report both absolute and relative effects of these models.

#### Moderators

We will use signal detection analysis [132] to assess whether there are any moderators or predictors of ≥5% weight loss at 6 months and whether this varies by self-monitoring strategy. Signal detection analysis is a nonparametric recursive partitioning technique that can identify patient subgroups that demonstrate a better or worse response to an intervention. In this analysis, we will examine demographic, psychosocial, behavioral, and clinical characteristics, all assessed at baseline. These exploratory analyses help generate hypotheses that can inform which interventions are most suitable for which individuals [133]. We will use publicly available ROC5 program software (Stanford University) to conduct this analysis [134].

#### Engagement and Its Association with Weight Loss

We will first assess patterns of self-monitoring engagement over 6 months using descriptive statistics (medians and IQR if non-normally distributed, as is expected [36]) and via graphical presentation. Then, we will use Spearman rank correlation coefficients (if engagement data are non-normally distributed) and linear regression models to examine the relation between self-monitoring engagement and weight change at 6 months. Using descriptive statistics, we will also assess completion of the other intervention components (action plans completed, lessons read, and progress reports reviewed) and follow similar procedures to examine the relation between engagement in each of these components and weight change at 6 months.

#### Characterizing the Sample

Descriptive statistics will be used to describe baseline characteristics. To determine whether any baseline variables differ by retention status (completers vs noncompleters), we will use Pearson chi-square tests for categorical variables, ANOVA for continuous variables, and Fisher exact tests for small cell counts.

#### Analytic Plan for the Embedded Experiment

We will assess differences in 6-month trial retention between participants who were randomized to receive versus not receive the self-directed orientation. We will fit a log-Poisson GEE model with robust SEs [129,130] and an unstructured working correlation matrix to take into account repeated measures across time. As is common in embedded experiments, sample size is constrained by the number of participants in the parent trial (the Spark weight loss trial) [66]. A power calculation was run to determine the effect size that could be detected assuming a sample size of 176, 80% power, an α of 5% in a 2-sided test, and retention of 65% in the arm not receiving the orientation (assuming 35% dropout). We would have 80% power to detect a retention rate of 83% in the arm receiving the orientation.

### Data Monitoring

The PI was responsible for regular monitoring of the data, in accordance with the Data Safety and Monitoring Plan. Issues of safety were reviewed with the independent safety monitor, who was not involved in the study’s design or conduct. If they occurred, major protocol deviations were reviewed by all investigators. The PI and study statistician will have access to the final dataset.

### Decision Criteria for Selecting Components for an Optimized Intervention

Using the MOST framework for intervention optimization, this study seeks to balance intervention effectiveness (ie, weight loss) against efficiency (ie, minimizing participant burden). Thus, our “Optimization Objective” is to create an intervention made up of all active components without any inactive components. To decide which self-monitoring strategies should comprise our newly optimized intervention, we will use the component screening approach described by Collins [54], which iteratively sorts through the results of the factorial trial. A minimum mean weight loss threshold of 2.1 (SD 4.5) kg at 6 months will be used to decide whether to consider including any self-monitoring components alone or in combination. This magnitude equates to an α of .10. We selected a higher alpha level during this decision-making phase when constructing the newly optimized intervention because we want to lower the chance of mistakenly discarding an intervention component that is actually beneficial (ie, reduce the type 2 error rate). This rationale reflects the “decision-priority perspective” of MOST. In contrast, the “conclusion-priority perspective” is focused on drawing scientific conclusions from a trial using well-established conventions of α level ≤.05 [54,55].

Using the component screening approach, first, the main effects of each self-monitoring component on 6-month weight change will be determined. The self-monitoring strategies that meet or exceed this threshold will be tentatively included in the “screened-in” set while those not meeting this threshold will be included in the “screened-out” set. Then, lower-level interactions will be examined, first among any components in the screened-in set, followed by those in the screened-out set. Next, we will examine the interaction among all 3 self-monitoring components. For interactions meeting or exceeding the 2.1 kg threshold of weight loss, their components will be considered for inclusion in the screened-in set (even if the components, by themselves, did not demonstrate main effects). To aid in interpretation of interactions, we will plot the predicted marginal means. This visualization will provide information on whether synergistic or antagonistic interactions exist. Results of this trial will inform development of a newly optimized intervention that can be tested in a subsequent evaluation-RCT. If no self-monitoring strategies, alone or in combination, exceed our 2.1 kg weight loss threshold, then we will return to the preparation phase of the MOST framework to refine the conceptual model, brainstorm and pilot test new candidate components, adapt existing components, or strengthen the core digital health intervention.

### Ethical Considerations

All study procedures and human subjects research ethics were approved by the Stanford University Institutional Review Board (protocol number 64716; approval date: March 28, 2022). The participants provided written informed consent via electronic signature before enrollment. They were informed that participation is voluntary, and they could withdraw or opt out at any time. The consent form is available on the trial registry [57]. Participants were compensated a maximum of US $60 (via electronic gift cards) for their completion of assessments, as follows: US $20 at 3 months, US $30 at 6 months, and an additional US $10 for completion of all 4 dietary recalls (2 each at baseline and 6 months). Those who participated in the qualitative interviews received an additional US $25. Deidentified data will be used in study analyses and in disseminated materials.

## Results

The study received funding in April 2022 (see Multimedia Appendix 2). The first participant was enrolled (ie, consented) in the Spark trial on September 22, 2023. Randomization to the factorial trial occurred between October 6, 2023 and November 26, 2024. We recruited 176 participants. Data collection was completed June 23, 2025. Upon analysis of trial data, results will be disseminated to study participants via an optional webinar. They will also be shared through presentations at national conferences, via publication in peer-reviewed journals, and posted on ClinicalTrials.gov. Data analysis is ongoing and results are expected to be published in early 2026.

## Discussion

### Scientific Contribution

The Spark trial will provide the first set of evidence on optimizing self-monitoring in a weight loss intervention for adults with overweight or obesity. It is common for behavioral obesity treatment to include self-monitoring of diet, physical activity, and body weight, yet it is not actually known whether all 3 of these components are necessary for weight loss or whether any are ineffective, or even detrimental. Removing any inactive components would help to minimize patient burden and effort. Using an intervention optimization framework (MOST) enables our team to build an effective and efficient fully digital weight loss intervention, which is much needed given the high prevalence of obesity, its detrimental health consequences, and the limited scalability of existing weight loss interventions. Further, to our knowledge, our trial will be the first to empirically test the impact of a self-directed web-based orientation session on trial retention. If deemed effective, this orientation session may be an affordable, brief, and scalable strategy that could be easily adapted and embedded in behavioral intervention research to enhance the validity of trial outcomes.

### Limitations

Several limitations exist. First, while our trial’s primary outcome is weight loss, it is possible that a component may have limited impact on weight but still improve diet quality, physical activity, or health-related quality of life. The MOST framework provides flexibility in interpreting the data in different ways depending on the Optimization Objective of interest. Thus, the intervention could be optimized for a different outcome, which would involve simply reinterpreting the trial’s existing data in light of that outcome. Second, due to funding constraints, we are unable to collect data beyond the 6-month time point, thus precluding the examination of long-term weight loss maintenance, which could be a focus of future research. Third, it is unknown whether results would generalize to digital weight loss interventions that include direct human counseling. However, by design, our trial focused on fully digital interventions (without such counseling) since they offer an opportunity to reach broad populations, including those with limited access to high-quality obesity treatment, and to deliver a weight loss program in a more scalable manner. Fourth, due to the behavioral focus on the intervention, participants are not masked to factorial condition, which could introduce bias. We aimed to establish clinical equipoise during our discussion of the various self-monitoring components at the baseline visit. Fifth, due to technical limitations, the Fitbit app is not able to reflect participants’ adaptive step goal each week. Therefore, the step goal feedback provided by the app likely differed than the feedback provided via our weekly progress reports. Sixth, our embedded experiment that is testing the effect of a self-directed orientation session may be underpowered to detect small differences in retention due to sample size constraints of the Spark trial. This is an inherent limitation of embedded experiments [67], yet given their simpler and less resource-intensive nature, they are designed to be replicated across multiple clinical trials to build up the evidence base on potential retention-promoting strategies. These data can then be evaluated together in meta-analyses.

### Implications for Research and Clinical Care

From a research standpoint, we hope that our findings will shed light on which self-monitoring strategies should be included in behavioral obesity treatments. Considering MOST’s continual optimization principle, interventions should be continuously improved upon in an iterative fashion to enhance outcomes. Future research could seek to optimize other components of fully digital interventions, such as type of feedback, skills training materials, and gamification approaches, and could test artificial intelligence–driven strategies for tailoring intervention content based on participant preferences, needs, and treatment response. From a clinical standpoint, we hope this work will inform clinicians who provide obesity counseling as to which self-monitoring strategies they should be recommending to their patients who are seeking to lose weight in a standalone manner.

### Conclusions

The Spark trial leverages an intervention optimization framework to understand whether self-monitoring diet, steps, or body weight maximizes weight loss, alone or in combination, which addresses a critical research gap. Ultimately, building a fully digital intervention comprised of only clinically meaningful self-monitoring strategies has potential for broad public health impact in providing scalable, potent, low burden, and far-reaching weight loss interventions. If the optimized intervention is effective, it could serve as a first-line weight loss treatment for adults with overweight or obesity.

## Appendix

Multimedia Appendix 1 SPIRIT 2025 checklist for the Spark trial.

Multimedia Appendix 2 Peer review report from the Digestive Diseases and Nutrition C Study Section Digestive Diseases and Nutrition DDK-C Subcommittee (National Institutes of Health, USA).

## Abbreviations

ASA24: Automated Self-Administered 24-hour
CONSORT: Consolidated Standards Of Reporting Trials
COREQ: Consolidated Criteria for Reporting Qualitative Research
GEE: generalized estimating equation
GLTEQ: Godin Leisure-Time Exercise Questionnaire
MOST: Multiphase Optimization Strategy
NHANES: National Health and Nutrition Examination Survey
PAR-Q+: Physical Activity Readiness Questionnaire for Everyone
PI: principal investigator
RCT: randomized clinical trial
REDCap: Research Electronic Data Capture
SF-36: 36-item Short Form Health Survey
SPIRIT: Standard Protocol Items: Recommendations for Interventional Trials
SWAT: Study Within a Trial

## Data Availability

Data sharing is not applicable to this article as no datasets were analyzed yet during this study.

## References

[1] Emmerich SD, Fryar CD, Stierman B, Ogden CL (2024). Obesity and severe obesity prevalence in adults: United States, August 2021-August 2023. Centers for Disease Control and Prevention.

[2] Fryar CC, Carroll MD, Afful J Prevalence of overweight, obesity, and severe obesity among adults aged 20 and over: United States, 1960–1962 through 2017–2018. NCHS Health E-Stats 2020.

[3] Curry SJ, Krist AH, Owens DK, Barry MJ, Caughey AB, Davidson KW, Doubeni CA, Epling JW, Grossman DC, Kemper AR, Kubik M, Landefeld CS, Mangione CM, Phipps MG, Silverstein M, Simon MA, Tseng C, Wong JB, US Preventive Services Task Force (2018). Behavioral weight loss interventions to prevent obesity-related morbidity and mortality in adults: US preventive services task force recommendation statement. JAMA.

[4] Wadden TA, Tronieri JS, Butryn ML (2020). Lifestyle modification approaches for the treatment of obesity in adults. Am Psychol.

[5] Anderson-Lewis C, Darville G, Mercado RE, Howell S, Di Maggio S (2018). mHealth technology use and implications in historically underserved and minority populations in the United States: systematic literature review. JMIR Mhealth Uhealth.

[6] Rosenbaum DL, Piers AD, Schumacher LM, Kase CA, Butryn ML (2017). Racial and ethnic minority enrollment in randomized clinical trials of behavioural weight loss utilizing technology: a systematic review. Obes Rev.

[7] Haughton CF, Silfee VJ, Wang ML, Lopez-Cepero AC, Estabrook DP, Frisard C, Rosal MC, Pagoto SL, Lemon SC (2018). Racial/ethnic representation in lifestyle weight loss intervention studies in the United States: a systematic review. Prev Med Rep.

[8] Ariel-Donges A, Gordon E, Dixon B, Eastman AJ, Bauman V, Ross KM, Perri MG (2020). Rural/urban disparities in access to the national diabetes prevention program. Transl Behav Med.

[9] (2020). Obesity and overweight. Centers for Disease Control and Prevention.

[10] Hales CM, Fryar CD, Carroll MD, Freedman DS, Aoki Y, Ogden CL (2018). Differences in obesity prevalence by demographic characteristics and urbanization level among adults in the United States, 2013-2016. JAMA.

[11] Singh B, Ahmed M, Staiano AE, Gough C, Petersen J, Vandelanotte C, Kracht C, Huong C, Yin Z, Vasiloglou MF, Pan C, Short CE, Mclaughlin M, von Klinggraeff L, Pfledderer CD, Moran LJ, Button AM, Maher CA (2024). A systematic umbrella review and meta-meta-analysis of eHealth and mHealth interventions for improving lifestyle behaviours. NPJ Digit Med.

[12] Islam MM, Poly TN, Walther BA, Jack Li Y (2020). Use of mobile phone app interventions to promote weight loss: meta-analysis. JMIR Mhealth Uhealth.

[13] Antoun J, Itani H, Alarab N, Elsehmawy A (2022). The effectiveness of combining nonmobile interventions with the use of smartphone apps with various features for weight loss: systematic review and meta-analysis. JMIR Mhealth Uhealth.

[14] Tang JCH, Abraham C, Greaves CJ, Nikolaou V (2016). Self-directed interventions to promote weight loss: a systematic review and meta-analysis. Health Psychol Rev.

[15] Unick JL, Pellegrini CA, Dunsiger SI, Demos KE, Thomas JG, Bond DS, Lee RH, Webster J, Wing RR (2024). An adaptive telephone coaching intervention for patients in an online weight loss program: a randomized clinical trial. JAMA Netw Open.

[16] Thomas JG, Panza E, Goldstein CM, Hayes JF, Benedict N, O'Leary K, Wing RR (2024). Pragmatic implementation of online obesity treatment and maintenance interventions in primary care: a randomized clinical trial. JAMA Intern Med.

[17] Spring B, Pfammatter AF, Scanlan L, Daly E, Reading J, Battalio S, McFadden HG, Hedeker D, Siddique J, Nahum-Shani I (2024). An adaptive behavioral intervention for weight loss management: a randomized clinical trial. JAMA.

[18] Hoddinott P, O'Dolan C, Macaulay L, Dombrowski SU, Swingler J, Cotton S, Avenell A, Getaneh AM, Gray C, Hunt K, Kee F, MacLean A, McKinley M, Torrens C, Turner K, van der Pol M, MacLennan G (2024). Text messages with financial incentives for men with obesity: a randomized clinical trial. JAMA.

[19] Markkanen JO, Oikarinen N, Savolainen MJ, Merikallio H, Nyman V, Salminen V, Virkkula T, Karppinen P, Oinas-Kukkonen H, Hukkanen J (2024). Mobile health behaviour change support system as independent treatment tool for obesity: a randomized controlled trial. Int J Obes (Lond).

[20] Baker RC, Kirschenbaum DS (1993). Self-monitoring may be necessary for successful weight control. Behav Ther.

[21] Burke LE, Wang J, Sevick MA (2011). Self-monitoring in weight loss: a systematic review of the literature. J Am Diet Assoc.

[22] Patel ML, Wakayama LN, Bennett GG (2021). Self-monitoring via digital health in weight loss interventions: a systematic review among adults with overweight or obesity. Obesity (Silver Spring).

[23] Raber M, Liao Y, Rara A, Schembre SM, Krause KJ, Strong L, Daniel-MacDougall C, Basen-Engquist K (2021). A systematic review of the use of dietary self-monitoring in behavioural weight loss interventions: delivery, intensity and effectiveness. Public Health Nutr.

[24] Yen H, Chiu H (2019). The effectiveness of wearable technologies as physical activity interventions in weight control: a systematic review and meta-analysis of randomized controlled trials. Obes Rev.

[25] Larsen RT, Wagner V, Korfitsen CB, Keller C, Juhl CB, Langberg H, Christensen J (2022). Effectiveness of physical activity monitors in adults: systematic review and meta-analysis. BMJ.

[26] Zheng Y, Klem ML, Sereika SM, Danford CA, Ewing LJ, Burke LE (2015). Self-weighing in weight management: a systematic literature review. Obesity (Silver Spring).

[27] Bandura A (1991). Social cognitive theory of self-regulation. Organ Behav Hum Decis Process.

[28] Carver CS, Scheier MF (1982). Control theory: a useful conceptual framework for personality-social, clinical, and health psychology. Psychol Bull.

[29] Michie S, Abraham C, Whittington C, McAteer J, Gupta S (2009). Effective techniques in healthy eating and physical activity interventions: a meta-regression. Health Psychol.

[30] Harkin B, Webb TL, Chang BPI, Prestwich A, Conner M, Kellar I, Benn Y, Sheeran P (2016). Does monitoring goal progress promote goal attainment? A meta-analysis of the experimental evidence. Psychol Bull.

[31] Dombrowski S, Sniehotta F, Avenell A, Johnston M, MacLennan G, Araújo-Soares V (2010). Identifying active ingredients in complex behavioural interventions for obese adults with obesity-related co-morbidities or additional risk factors for co-morbidities: a systematic review. Health Psychol Rev.

[32] Krukowski RA, Denton AH, König LM (2024). Impact of feedback generation and presentation on self-monitoring behaviors, dietary intake, physical activity, and weight: a systematic review and meta-analysis. Int J Behav Nutr Phys Act.

[33] Hartmann-Boyce J, Johns DJ, Jebb SA, Aveyard P, Behavioural Weight Management Review Group (2014). Effect of behavioural techniques and delivery mode on effectiveness of weight management: systematic review, meta-analysis and meta-regression. Obes Rev.

[34] Berry R, Kassavou A, Sutton S (2021). Does self-monitoring diet and physical activity behaviors using digital technology support adults with obesity or overweight to lose weight? A systematic literature review with meta-analysis. Obes Rev.

[35] Cavero-Redondo I, Martinez-Vizcaino V, Fernandez-Rodriguez R, Saz-Lara A, Pascual-Morena C, Álvarez-Bueno C (2020). Effect of behavioral weight management interventions using lifestyle mHealth self-monitoring on weight loss: a systematic review and meta-analysis. Nutrients.

[36] Patel ML, Hopkins CM, Brooks TL, Bennett GG (2019). Comparing self-monitoring strategies for weight loss in a smartphone app: randomized controlled trial. JMIR Mhealth Uhealth.

[37] Turner-McGrievy GM, Dunn CG, Wilcox S, Boutté AK, Hutto B, Hoover A, Muth E (2019). Defining adherence to mobile dietary self-monitoring and assessing tracking over time: tracking at least two Eating Occasions per day is best marker of adherence within two different mobile health randomized weight loss interventions. J Acad Nutr Diet.

[38] Thomas JG, Bond DS, Raynor HA, Papandonatos GD, Wing RR (2019). Comparison of smartphone-based behavioral obesity treatment with gold standard group treatment and control: a randomized trial. Obesity (Silver Spring).

[39] Butryn ML, Martinelli MK, Crane NT, Godfrey K, Roberts SR, Zhang F, Forman EM (2020). Counselor surveillance of digital self-monitoring data: a pilot randomized controlled trial. Obesity (Silver Spring).

[40] Turner-McGrievy G, Yang C, Monroe C, Pellegrini C, West D (2021). Is burden always bad? Emerging low-burden approaches to mobile dietary self-monitoring and the role burden plays with engagement. J Technol Behav Sci.

[41] Burke LE, Swigart V, Warziski Turk M, Derro N, Ewing LJ (2009). Experiences of self-monitoring: successes and struggles during treatment for weight loss. Qual Health Res.

[42] Li S, Du Y, Meireles C, Song D, Sharma K, Yin Z, Brimhall B, Wang J (2024). Decoding heterogeneity in data-driven self-monitoring adherence trajectories in digital lifestyle interventions for weight loss: a qualitative study. BMC Digit Health.

[43] Steinberg DM, Tate DF, Bennett GG, Ennett S, Samuel-Hodge C, Ward DS (2013). The efficacy of a daily self-weighing weight loss intervention using smart scales and e-mail. Obesity (Silver Spring).

[44] Kaviani S, vanDellen M, Cooper JA (2019). Daily self-weighing to prevent holiday-associated weight gain in adults. Obesity (Silver Spring).

[45] Jospe MR, Roy M, Brown RC, Williams SM, Osborne HR, Meredith-Jones KA, McArthur JR, Fleming EA, Taylor RW (2017). The effect of different types of monitoring strategies on weight loss: a randomized controlled trial. Obesity (Silver Spring).

[46] Saslow LR, Missel AL, O'Brien A, Kim S, Hecht FM, Moskowitz JT, Bayandorian H, Pietrucha M, Raymond K, Richards B, Liestenfeltz B, Mason AE, Daubenmier J, Aikens JE (2023). Psychological support strategies for adults with type 2 diabetes in a very low-carbohydrate web-based program: randomized controlled trial. JMIR Diabetes.

[47] Patel ML, Cleare AE, Smith CM, Rosas LG, King AC (2022). Detailed versus simplified dietary self-monitoring in a digital weight loss intervention among racial and ethnic minority adults: fully remote, randomized pilot study. JMIR Form Res.

[48] Nezami BT, Hurley L, Power J, Valle CG, Tate DF (2022). A pilot randomized trial of simplified versus standard calorie dietary self-monitoring in a mobile weight loss intervention. Obesity (Silver Spring).

[49] Dunn CG, Turner-McGrievy GM, Wilcox S, Hutto B (2019). Dietary self-monitoring through calorie tracking but not through a digital photography app is associated with significant weight loss: the 2SMART pilot study-A 6-month randomized trial. J Acad Nutr Diet.

[50] Turner-McGrievy GM, Wilcox S, Boutté A, Hutto BE, Singletary C, Muth ER, Hoover AW (2017). The dietary intervention to enhance tracking with mobile devices (DIET Mobile) study: a 6-month randomized weight loss trial. Obesity (Silver Spring).

[51] Helsel DL, Jakicic JM, Otto AD (2007). Comparison of techniques for self-monitoring eating and exercise behaviors on weight loss in a correspondence-based intervention. J Am Diet Assoc.

[52] Pagoto S, Tulu B, Waring ME, Goetz J, Bibeau J, Divito J, Groshon L, Schroeder M (2021). Slip buddy app for weight management: randomized feasibility trial of a dietary lapse tracking app. JMIR Mhealth Uhealth.

[53] Berry MP (2023). A randomized pilot trial of a tailored, reduced-frequency approach to dietary self-monitoring for weight loss. Drexel University.

[54] Collins LM (2018). Optimization of Behavioral, Biobehavioral, and Biomedical Interventions.

[55] Collins LM, Nahum-Shani I, Guastaferro K, Strayhorn JC, Vanness DJ, Murphy SA (2024). Intervention optimization: a paradigm shift and its potential implications for clinical psychology. Annu Rev Clin Psychol.

[56] Chan A, Boutron I, Hopewell S, Moher D, Schulz KF, Collins GS, Tunn R, Aggarwal R, Berkwits M, Berlin JA, Bhandari N, Butcher NJ, Campbell MK, Chidebe RCW, Elbourne DR, et al (2025). SPIRIT 2025 statement: updated guideline for protocols of randomized trials. JAMA.

[57] Patel ML (2023). Spark: finding the optimal tracking strategy for weight loss in a digital health intervention. ClinicalTrials.gov.

[58] Patel ML (2023). Ignite pilot: goal setting in a digital weight loss intervention. ClinicalTrials.gov.

[59] Jensen MD, Ryan DH, Apovian CM, Ard JD, Comuzzie AG, Donato KA, Hu FB, Hubbard VS, Jakicic JM, Kushner RF, Loria CM, Millen BE, Nonas CA, Pi-Sunyer FX, Stevens J, et al VJ (2014). 2013 AHA/ACC/TOS guideline for the management of overweight and obesity in adults: a report of the American college of cardiology/American heart association task force on practice guidelines and the obesity society. Circulation.

[60] Warburton DE, Jamnik VK, Bredin SS, Gledhill N (2011). The physical activity readiness questionnaire for everyone (PAR-Q+) and electronic physical activity readiness medical examination (ePARmed-X+). The Health & Fitness Journal of Canada.

[61] Hales CM, Carroll MD, Fryar CD, Ogden CL (2020). Prevalence of obesity and severe obesity among adults: United States, 2017-2018. NCHS Data Brief.

[62] Harris PA, Taylor R, Minor BL, Elliott V, Fernandez M, O'Neal L, McLeod L, Delacqua G, Delacqua F, Kirby J, Duda SN, REDCap Consortium (2019). The REDCap consortium: building an international community of software platform partners. J Biomed Inform.

[63] Patel ML (2023). SWAT 211: effects of a self-directed orientation session on retention in a digital weight loss study. The Northern Ireland Network for Trials Methodology Research.

[64] Jake-Schoffman DE, Brown SD, Baiocchi M, Bibeau JL, Daubenmier J, Ferrara A, Galarce MN, Hartogensis W, Hecht FM, Hedderson MM, Moran PJ, Pagoto SL, Tsai A, Waring ME, Kiernan M (2021). Methods-motivational interviewing approach for enhanced retention and attendance. Am J Prev Med.

[65] Goldberg JH, Kiernan M (2005). Innovative techniques to address retention in a behavioral weight-loss trial. Health Educ Res.

[66] Treweek S, Bevan S, Bower P, Campbell M, Christie J, Clarke M, Collett C, Cotton S, Devane D, El Feky A, Flemyng E, Galvin S, Gardner H, Gillies K, Jansen J, Littleford R, Parker A, Ramsay C, Restrup L, Sullivan F, Torgerson D, Tremain L, Westmore M, Williamson PR (2018). Trial forge guidance 1: what is a study within A trial (SWAT)?. Trials.

[67] Parker A, Arundel C, Clark L, Coleman E, Doherty L, Hewitt CE, Beard D, Bower P, Cooper C, Culliford L, Devane D, Emsley R, Eldridge S, Galvin S, Gillies K, Montgomery A, Sutton CJ, Treweek S, Torgerson DJ (2024). Undertaking studies within A trial to evaluate recruitment and retention strategies for randomised controlled trials: lessons learnt from the PROMETHEUS research programme. Health Technol Assess.

[68] Gallis JA, Bennett GG, Steinberg DM, Askew S, Turner EL (2019). Randomization procedures for multicomponent behavioral intervention factorial trials in the multiphase optimization strategy framework: challenges and recommendations. Transl Behav Med.

[69] Cleland C (2018). REDCap with MOST. Cadio.

[70] (2018). PreventT2 curriculum and handouts. US Centers for Disease Control and Prevention.

[71] Berry MP, Chwyl C, Metzler AL, Sun JH, Dart H, Forman EM (2023). Associations between behaviour change technique clusters and weight loss outcomes of automated digital interventions: a systematic review and meta-regression. Health Psychol Rev.

[72] Carraça E, Encantado J, Battista F, Beaulieu K, Blundell J, Busetto L, van Baak M, Dicker D, Ermolao A, Farpour-Lambert N, Pramono A, Woodward E, Bellicha A, Oppert J (2021). Effective behavior change techniques to promote physical activity in adults with overweight or obesity: a systematic review and meta-analysis. Obes Rev.

[73] Hawkins LK, Burns L, Swancutt D, Moghadam S, Pinkney J, Tarrant M, PROGROUP Programme Team (2024). Which components of behavioral weight management programs are essential for weight loss in people living with obesity? A rapid review of systematic reviews. Obes Rev.

[74] Schembre SM, Liao Y, Robertson MC, Dunton GF, Kerr J, Haffey ME, Burnett T, Basen-Engquist K, Hicklen RS (2018). Just-in-time feedback in diet and physical activity interventions: systematic review and practical design framework. J Med Internet Res.

[75] Marques MM, Wright AJ, Corker E, Johnston M, West R, Hastings J, Zhang L, Michie S (2023). The behaviour change technique ontology: transforming the behaviour change technique taxonomy v1. Wellcome Open Res.

[76] Miller WR, Rollnick S (2012). Motivational Interviewing: Helping People Change.

[77] Patel ML, Wakayama LN, Bass MB, Breland JY (2019). Motivational interviewing in eHealth and telehealth interventions for weight loss: a systematic review. Prev Med.

[78] Perri MG, Nezu AM, McKelvey WF, Shermer RL, Renjilian DA, Viegener BJ (2001). Relapse prevention training and problem-solving therapy in the long-term management of obesity. J Consult Clin Psychol.

[79] Sherrington A, Newham JJ, Bell R, Adamson A, McColl E, Araujo-Soares V (2016). Systematic review and meta-analysis of internet-delivered interventions providing personalized feedback for weight loss in overweight and obese adults. Obes Rev.

[80] Madigan CD, Daley AJ, Lewis AL, Aveyard P, Jolly K (2015). Is self-weighing an effective tool for weight loss: a systematic literature review and meta-analysis. Int J Behav Nutr Phys Act.

[81] Burke LE, Conroy MB, Sereika SM, Elci OU, Styn MA, Acharya SD, Sevick MA, Ewing LJ, Glanz K (2011). The effect of electronic self-monitoring on weight loss and dietary intake: a randomized behavioral weight loss trial. Obesity (Silver Spring).

[82] Mifflin M, St Jeor S, Hill L, Scott B, Daugherty S, Koh Y (1990). A new predictive equation for resting energy expenditure in healthy individuals. Am J Clin Nutr.

[83] Godin SR (1997). Godin leisure-time exercise questionnaire. Med Sci Sports Exerc.

[84] Godin G, Shephard RJ (1985). A simple method to assess exercise behavior in the community. Can J Appl Sport Sci.

[85] Adams MA, Sallis JF, Norman GJ, Hovell MF, Hekler EB, Perata E (2013). An adaptive physical activity intervention for overweight adults: a randomized controlled trial. PLoS One.

[86] Adams MA, Todd M, Angadi SS, Hurley JC, Stecher C, Berardi V, Phillips CB, McEntee ML, Hovell MF, Hooker SP (2022). Adaptive goals and reinforcement timing to increase physical activity in adults: a factorial randomized trial. Am J Prev Med.

[87] Ross KM, Wing RR (2016). Concordance of in-home "Smart" scale measurement with body weight measured in-person. Obes Sci Pract.

[88] Pebley K, Klesges RC, Talcott GW, Kocak M, Krukowski RA (2019). Measurement equivalence of E-scale and in-person clinic weights. Obesity (Silver Spring).

[89] Cui Z, Stevens J, Truesdale KP, Zeng D, French S, Gordon-Larsen P (2016). Prediction of body mass index using concurrently self-reported or previously measured height and weight. PLoS One.

[90] Subar AF, Kirkpatrick SI, Mittl B, Zimmerman TP, Thompson FE, Bingley C, Willis G, Islam NG, Baranowski T, McNutt S, Potischman N (2012). The automated self-administered 24-hour dietary recall (ASA24): a resource for researchers, clinicians, and educators from the national cancer institute. J Acad Nutr Diet.

[91] Shams-White MM, Pannucci TE, Lerman JL, Herrick KA, Zimmer M, Meyers Mathieu K, Stoody EE, Reedy J (2023). Healthy eating index-2020: review and update process to reflect the dietary guidelines for Americans,2020-2025. J Acad Nutr Diet.

[92] Kiernan M, Schoffman DE, Lee K, Brown SD, Fair JM, Perri MG, Haskell WL (2013). The stanford leisure-time activity categorical item (L-Cat): a single categorical item sensitive to physical activity changes in overweight/obese women. Int J Obes (Lond).

[93] Linde JA, Rothman AJ, Baldwin AS, Jeffery RW (2006). The impact of self-efficacy on behavior change and weight change among overweight participants in a weight loss trial. Health Psychol.

[94] Ware JE (2000). SF-36 health survey update. Spine (Phila Pa 1976).

[95] Clark MM, Abrams DB, Niaura RS, Eaton CA, Rossi JS (1991). Self-efficacy in weight management. J Consult Clin Psychol.

[96] Sallis JF, Pinski RB, Grossman RM, Patterson TL, Nader PR (1988). The development of self-efficacy scales for healthrelated diet and exercise behaviors. Health Educ Res.

[97] Karlsson J, Persson L, Sjöström L, Sullivan M (2000). Psychometric properties and factor structure of the three-factor eating questionnaire (TFEQ) in obese men and women. Results from the Swedish obese subjects (SOS) study. Int J Obes Relat Metab Disord.

[98] Buysse DJ, Reynolds CF, Monk TH, Berman SR, Kupfer DJ (1989). The pittsburgh sleep quality Index: a new instrument for psychiatric practice and research. Psychiatry Res.

[99] Levesque CS, Williams GC, Elliot D, Pickering MA, Bodenhamer B, Finley PJ (2007). Validating the theoretical structure of the treatment self-regulation questionnaire (TSRQ) across three different health behaviors. Health Educ Res.

[100] Verplanken B, Orbell S (2006). Reflections on past behavior: A self-report index of habit strength1. J Appl Soc Psychol.

[101] Gardner B, Abraham C, Lally P, de Bruijn G (2012). Towards parsimony in habit measurement: testing the convergent and predictive validity of an automaticity subscale of the self-report habit index. Int J Behav Nutr Phys Act.

[102] Bandura A (2006). Guide for constructing self-efficacy scales. Self-efficacy beliefs of adolescents.

[103] Neff K, King A (1995). Exercise program adherence in older adults: the importance of achieving one?s expected benefits. Med Exerc Nutr Health.

[104] Weiss BD, Mays MZ, Martz W, Castro KM, DeWalt DA, Pignone MP, Mockbee J, Hale FA (2005). Quick assessment of literacy in primary care: the newest vital sign. Ann Fam Med.

[105] Weiss BD (2024). What's new about the newest vital sign?. Health Lit Res Pract.

[106] Durso LE, Latner JD (2008). Understanding self-directed stigma: development of the weight bias internalization scale. Obesity (Silver Spring).

[107] Cohen S, Kamarck T, Mermelstein R (1994). Perceived stress scale. Measuring Stress: A Guide for Health and Social Scientists.

[108] Andrés A, Saldaña C, Gómez-Benito J (2009). Establishing the stages and processes of change for weight loss by consensus of experts. Obesity (Silver Spring).

[109] Sallis JF, Grossman RM, Pinski RB, Patterson TL, Nader PR (1987). The development of scales to measure social support for diet and exercise behaviors. Prev Med.

[110] Krukowski RA, Ross KM (2020). Measuring weight with electronic scales in clinical and research settings during the coronavirus disease 2019 pandemic. Obesity (Silver Spring).

[111] (2016). Automated Self-Administered 24-Hour (ASA24) Dietary Assessment Tool. National Cancer Institute.

[112] Amireault S, Godin G (2015). The godin-shephard leisure-time physical activity questionnaire: validity evidence supporting its use for classifying healthy adults into active and insufficiently active categories. Percept Mot Skills.

[113] Yao J, Tan CS, Lim N, Tan J, Chen C, Müller-Riemenschneider F (2021). Number of daily measurements needed to estimate habitual step count levels using wrist-worn trackers and smartphones in 212,048 adults. Sci Rep.

[114] Germini F, Noronha N, Borg Debono V, Abraham Philip B, Pete D, Navarro T, Keepanasseril A, Parpia S, de Wit K, Iorio A (2022). Accuracy and acceptability of wrist-wearable activity-tracking devices: systematic review of the literature. J Med Internet Res.

[115] Li S, Du Y, Miao H, Sharma K, Li C, Yin Z, Brimhall B, Wang J (2024). Understanding heterogeneity in individual responses to digital lifestyle intervention through self-monitoring adherence trajectories in adults with overweight or obesity: secondary analysis of a 6-month randomized controlled trial. J Med Internet Res.

[116] Schumacher Leah M, Martinelli Mary K, Convertino Alexandra D, Forman Evan M, Butryn Meghan L (2021). Weight-Related Information Avoidance Prospectively Predicts Poorer Self-Monitoring and Engagement in a Behavioral Weight Loss Intervention. Ann Behav Med.

[117] Orstad SL, Gerchow L, Patel NR, Reddy M, Hernandez C, Wilson DK, Jay M (2021). Defining valid activity monitor data: A multimethod analysis of weight-loss intervention participants' barriers to wear and first 100 days of physical activity. Informatics (MDPI).

[118] Fetters MD, Curry LA, Creswell JW (2013). Achieving integration in mixed methods designs-principles and practices. Health Serv Res.

[119] Creswell JW, Creswell JD (2017). Research Design: Qualitative, Quantitative, and Mixed Methods Approaches.

[120] Guetterman TC, Fetters MD, Creswell JW (2015). Integrating quantitative and qualitative results in health science mixed methods research through joint displays. Ann Fam Med.

[121] Tong A, Sainsbury P, Craig J (2007). Consolidated criteria for reporting qualitative research (COREQ): a 32-item checklist for interviews and focus groups. Int J Qual Health Care.

[122] Collins LM, Dziak JJ, Kugler KC, Trail JB (2014). Factorial experiments: efficient tools for evaluation of intervention components. Am J Prev Med.

[123] Collins LM, Murphy SA, Strecher V (2007). The multiphase optimization strategy (MOST) and the sequential multiple assignment randomized trial (SMART): new methods for more potent eHealth interventions. Am J Prev Med.

[124] Crane MM, Lutes LD, Ward DS, Bowling JM, Tate DF (2015). A randomized trial testing the efficacy of a novel approach to weight loss among men with overweight and obesity. Obesity (Silver Spring).

[125] Thomas JG, Raynor HA, Bond DS, Luke AK, Cardoso CC, Wojtanowski AC, Vander Veur S, Tate D, Wing RR, Foster GD (2017). Weight loss and frequency of body-weight self-monitoring in an online commercial weight management program with and without a cellular-connected 'smart' scale: a randomized pilot study. Obes Sci Pract.

[126] Bennett GG, Steinberg D, Bolton J, Gallis JA, Treadway C, Askew S, Kay MC, Pollak KI, Turner EL (2021). Optimizing an obesity treatment using the multiphase optimization strategy framework: protocol for a randomized factorial trial. JMIR Res Protoc.

[127] Dziak J, Collins L, Wagner A (2013). FactorialPowerPlan SAS macro suite users? guide.

[128] Liang KY, Zeger SL (1986). Longitudinal data analysis using generalized linear models. Biometrika.

[129] Gallis JA, Turner EL (2019). Relative measures of association for binary outcomes: challenges and recommendations for the global health researcher. Ann Glob Health.

[130] Zou GY, Donner A (2013). Extension of the modified poisson regression model to prospective studies with correlated binary data. Stat Methods Med Res.

[131] Hopewell S, Chan A, Collins GS, Hróbjartsson A, Moher D, Schulz KF, Tunn R, Aggarwal R, Berkwits M, Berlin JA, Bhandari N, Butcher NJ, Campbell MK, Chidebe RCW, Elbourne D, et al (2025). CONSORT 2025 statement: updated guideline for reporting randomized trials. JAMA.

[132] Kraemer HC (1988). Assessment of 2 × 2 associations: generalization of signal-detection methodology. Am Stat.

[133] Kraemer HC, Wilson GT, Fairburn CG, Agras WS (2002). Mediators and moderators of treatment effects in randomized clinical trials. Arch Gen Psychiatry.

[134] Yesavage JA Signal detection software for receiver operator characteristics (ROC). Stanford University.

