# Supplementary material for: Optimizing Self-Monitoring in a Digital Weight Loss Intervention (Spark): Protocol for a Factorial Randomized Trial
Source: JMIR Res Protoc. 2025 Sep 23;14:e75629. doi: 10.2196/75629 (PMC12504904; doi:10.2196/75629)
Supplement: Multimedia Appendix 2 [file resprot_v14i1e75629_app2.pdf]

SUMMARY STATEMENT

PROGRAM CONTACT: (Privileged Communication) *Release Date:* 11/04/2021

Revised Date:

[redacted]

Application Number: 1 K23 DK129805-01A1

Principal Investigator *Formerly:* 1K23DK129805-01

PATEL, MICHELE LANPHER

Applicant Organization: STANFORD UNIVERSITY

Review Group: DDK-C
Digestive Diseases and Nutrition C Study Section
Digestive Diseases and Nutrition DDK-C Subcommittee

Meeting Date: 10/21/2021 *RFA/PA:* PA20-206
Council: JAN 2022 *PCC:* NVO CTTR
Requested Start: 04/01/2022

Project Title: Optimizing self-monitoring in a digital health intervention for weight loss

SRG Action: [redacted]
Next Steps: Visit https://grants.nih.gov/grants/next_steps.htm
Human Subjects: 30-Human subjects involved - Certified, no SRG concerns
Animal Subjects: 10-No live vertebrate animals involved for competing appl.
Gender: 1A-Both genders, scientifically acceptable
Minority: 1A-Minorities and non-minorities, scientifically acceptable
Age: 3A-No children included, scientifically acceptable

Project Year	Direct Costs Requested	Estimated Total Cost
1	179,000	193,320
2	179,000	193,320
3	179,000	193,320
4	179,000	193,320
5	179,000	193,320
TOTAL	895,000	966,600

ADMINISTRATIVE BUDGET NOTE: The budget shown is the requested budget and has not been adjusted to reflect any recommendations made by reviewers. If an award is planned, the costs will be calculated by Institute grants management staff based on the recommendations outlined below in the COMMITTEE BUDGET RECOMMENDATIONS section.

1K23DK129805-01A1 Patel, Michele

SCIENTIFIC REVIEW OFFICER'S NOTES

RESUME AND SUMMARY OF DISCUSSION: This application was resubmitted in response to program announcement PA-20-206, entitled “Mentored Patient-Oriented Research Career Development Award (K23 - Independent Clinical Trial Required)”. The proposed project aims to optimize a standalone digital intervention for weight loss by identifying which self- monitoring components (tracking diet, physical activity, or body weight), alone or in combination, maximize success while minimizing burden. As the discussion opened, the reviewers noted numerous strengths with the application including a very productive candidate who has adequately responded to the previous review regarding potential concerns with patients’ recruitment and retention and the vague institutional support. The revised application is improved. Additional strengths that were noticed previously such as a relevant topic of investigation; an outstanding training environment; a well thought out career development plan; and an excellent mentoring team, which includes mentors with complementary expertise remain. Despite these strengths, minor lingering concerns were raised regarding the limited power for evaluating the impact of individual self-monitoring components. Overall, this application is rated as outstanding-to-exceptional.

DESCRIPTION (provided by applicant): Behavioral obesity treatments can produce clinically significant weight loss but are often too costly or intensive to be implemented on a large scale. Standalone digital health interventions offer greater scalability than traditional in-person approaches, but produce only modest weight loss. To maximize efficacy, it is vital to determine the “active ingredients” of an intervention and eliminate the ineffective, or even detrimental, ones. Self-monitoring is a core component of behavioral obesity treatment that can be delivered via digital tools, yet little is known about the unique and combined impact of different self-monitoring strategies. The K23 candidate, Dr. Michele Patel, will address this gap by applying an innovative framework – the Multiphase Optimization Strategy (MOST) – to identify the most potent combination of digital self-monitoring strategies for weight loss. As the first part of this programmatic line of research, Dr. Patel will conduct a 6-month optimization trial that randomizes 176 adults with overweight/obesity to 0-3 self-monitoring components (tracking dietary intake, physical activity, and/or body weight) using a full factorial design. This study will leverage existing commercial platforms for self-monitoring, including a mobile app, wearable activity monitor, and wireless electronic scale. All participants will also receive an empirically- and theory-informed core weight loss intervention that includes goal setting, weekly tailored feedback, action plans, and behavioral skills training – components that enhance engagement and are well-supported by prior research. Aim 1a will examine the optimal combination of self-monitoring strategies that maximizes 6-month weight loss while Aim 1b will examine self-monitoring engagement and its association with weight loss. Aim 2 will evaluate barriers to and facilitators of engaging in these self-monitoring strategies, which will be assessed via semi-structured qualitative interviews with 40 trial participants. Aim 3 will assess a novel, interactive recruitment strategy via an embedded trial. Together, results will inform an R01 grant that evaluates the newly optimized intervention in an RCT. Building on Dr. Patel’s background in clinical trial methodology and behavioral obesity treatment, the proposed career development award will provide substantive training in 1) MOST and factorial designs; 2) qualitative and mixed methods research; 3) innovative recruitment and retention strategies; and 4) preparation for the transition into independent research. To facilitate successful completion of these goals, Stanford University’s outstanding environment for interdisciplinary research will be coupled with a highly qualified, well- rounded mentorship team comprised of Primary Mentor Dr. Abby King, Co-mentors Dr. Gary Bennett and Dr. Lisa Rosas, and Consultants Mr. John Gallis (biostatistician) and Dr. Linda Collins (developer of MOST). This K23 will position Dr. Patel to become a leader in optimizing digital interventions for weight loss and will launch her career as an independent investigator dedicated to treating obesity through innovative solutions.

PUBLIC HEALTH RELEVANCE: Creating an effective and scalable weight loss intervention is fundamental to treating obesity on a population level. This proposal seeks to optimize a standalone digital intervention for weight loss by identifying which self- monitoring components (tracking diet, physical activity, or body weight), alone or in combination, maximize success while minimizing burden. This line of work has potential for important public health impact by enhancing weight loss in digital treatments that can reach broad populations of adults with overweight/obesity.

CRITIQUES: The written critiques of individual reviewers are provided in essentially unedited form below. These critiques were prepared prior to the meeting and may not have been revised afterwards. The "RESUME AND SUMMARY OF DISCUSSION" above summarizes the final opinions of the committee.

CRITIQUE 1

Candidate: 1
Career Development Plan/Career Goals /Plan to Provide Mentoring: 1
Research Plan: 2
Mentor(s), Co-Mentor(s), Consultant(s), Collaborator(s): 1
Environment Commitment to the Candidate: 2

Overall Impact: This is a revised K23 application from a T32 postdoctoral fellow at the Stanford Prevention Research Center. The candidate has been fairly productive: she has published 15 peer reviewed manuscripts with 6 as first author and as a graduate student, she received several awards for her research including the Outstanding Dissertation Award from the Society of Behavioral Medicine and The Obesity Society's Master's Thesis award. She has also received several external and internal small grants to fund her research to date. The mentorship team and environment are excellent and will [redacted]rovide a valuable trainin[redacted] environment for the candidate. [redacted]

The candidate proposes 4 training aims: 1) MOST and factorial designs; 2) qualitative and mixed methods research; 3) innovative recruitment and retention strategies; and 4) preparation for the transition into independent research. The training aims are broadly relevant to the candidates short-term and long-term career goals and build off of her prior research background. The training plan consists of a mix of didactic and mentorship-based activities. On first submission she received feedback about her training plan being somewhat vague about what types of hands-on activities she would complete with mentors. She has now provided more detailed training plans and now lists a number of manuscripts she plans to write with her mentorship team. Of note, the candidate was highly responsive to past reviews suggesting concerns with recruitment/retention for a relatively low intensity study and has now added a full training aim devoted to innovative recruitment and retention strategies. She also recently pursued and was awarded funding ($16,800) to test novel ways to recruit racial/ethnic minority groups in a digital weight loss intervention. The new pilot data and new training aims related to recruitment and retention for racial/ethnic minorities and for digital health interventions is highly responsive and innovative.

The candidate proposes to conduct a 6-month pilot optimization trial that randomizes 176 adults with overweight/obesity to 0 to 3 self-monitoring components (tracking dietary intake, physical activity, and/or body weight) using a full factorial design. This study will leverage existing commercial platforms for self-monitoring, including a mobile app, wearable activity monitor, and wireless electronic scale. All participants will also receive an empirically- and theory-informed core weight loss intervention that

includes goal setting, weekly tailored feedback, action plans, and behavioral skills training – components that enhance engagement and are well-supported by prior research. Aim 1a will examine the optimal combination of self-monitoring strategies that maximizes 6-month weight loss while Aim 1b will examine self-monitoring engagement and its association with weight loss. Aim 2 will evaluate barriers to and facilitators of engaging in these self-monitoring strategies, which will be assessed via semi-structured qualitative interviews with 40 trial participants. Aim 3 will assess a novel, interactive recruitment strategy via an embedded trial. The study has many strengths including high scientific rigor, the used of a mixed methods design, the use of an ambitious MOST factorial design, and high significance. The candidate previously received some concerns about potential high attrition from a digital health study and in response to that concern has now increased her sample size to 176 to allow for up to 35% attrition. She also now proposes to examine synergistic effects of intervention components which was not included in her study previously and was noted as a limitation in past reviews. The addition of Aim 3, where she will test a novel retention tool (i.e., an interactive orientation video) in an RCT embedded in my larger trial is an entirely new aim, is consistent with her new training aims, and is also responsive to past reviews where she received feedback that the project was insufficiently large in scope for a 5-year award. Overall, the candidate has been highly responsive to past reviews and the grant application is excellent.

1. Candidate

Strengths

- The candidate is currently on a T32 postdoctoral fellowship at the Stanford Prevention Research Center
- She has published 15 peer reviewed manuscripts with 6 as first author
- As a graduate student, she received several awards for her research including the Outstanding Dissertation Award from the Society or Behavioral Medicine and The Obesity Society's Master's Thesis award.
- Has been successful in obtaining research funds through both national (American Psychological Association's Dissertation Research Award) and internal grants (e.g., Stanford's SPECTRUM Pilot Grant).

Weaknesses

- None noted

2. Career Development Plan/Career Goals & Objectives

[redacted]

3. Research Plan

Strengths

- Creating highly disseminable/low intensity but effective digital self-help programs for obesity is a significant area.
- This study will be the first to test the independent efficacy and engagement with multiple types of self-monitoring during a digital program for obesity.
- Use of the MOST strategy will allow for efficient way to examine the effects of each self-monitoring component with adequate power.
- The use of a mixed methods design will provide additional direction to inform future R01 research in this area.
- Stratification of weight loss outcomes for the qualitative study is a strength.
- Overall, the study has strong methodological rigor
- The candidate previously received some concerns about potential high attrition from a digital health study and in response to that concern has now increased her sample size to 176 to allow for up to 35% attrition.
- The addition of Aim 3, where she will test a novel retention tool (i.e., an interactive orientation video) in an RCT embedded in my larger trial is innovative and will set her up well to be successful in future R01 level projects.

Weaknesses

- There are fairly minimal details provided about the Methods-Motivational Interviewing approach that will be used in the retention video or any data demonstrating that this has proven effective in other trials that have used this approach.

4. Mentor(s), Co-Mentor(s), Consultant(s), Collaborator(s)

Strengths

- The Primary Mentor is Abby King, PhD an internationally renowned leader in physical activity and health behavior changes who will provide training in physical activity intervention and assessment practices and provide overall career development mentorship.
- Co-mentor: Dori Steinberg, PhD, an expert in designing digital health interventions for dietary change and chronic disease management,
- Co-mentor: Lisa Rosas, PhD, MPH is an expert in applying mixed methods approaches to behavioral interventions for preventing chronic disease.
- Consultant: Linda Collins, PhD, the developer of the Multiphase Optimization Strategy (MOST), will consult on using MOST to design and implement my pilot optimization trial, as well as decision-making to construct my R01 evaluating a fully-powered optimization trial.
- Consultant: John Gallis, ScM will provide consultation on analyzing outcomes from the trial
- The mentorship team all has significant ongoing funding in relevant areas that will provide the candidate valuable training activities.

Weaknesses

- None noted

5. Environment and Institutional Commitment to the Candidate

Strengths

- The Stanford Prevention Research Center is an excellent location for the proposed project
- Institutional Commitment letter now states that she will be promoted to Instructor of Medicine (a faculty level position) upon completion of postdoctoral training, not conditional to receiving this award.

Weaknesses

None noted

Study Timeline:

Strengths

- Past data support the feasibility of recruitment goals in the timeline noted

Weaknesses

- None noted

Protections for Human Subjects

Acceptable Risks and Adequate Protections

- Acceptable risks for a low intensity study

Data and Safety Monitoring Plan (Applicable for Clinical Trials Only):

The candidate proposes to monitor safety in conjunction with the primary mentor. An independent safety officer would be beneficial- This feedback was previously given at the last review.

Inclusion Plans

- Sex/Gender: Distribution justified scientifically
- Race/Ethnicity: Distribution justified scientifically
- For NIH-Defined Phase III trials, Plans for valid design and analysis: Not applicable
- Inclusion/Exclusion Based on Age: Distribution justified scientifically
- Candidate will now recruit 50% racial/ethnic minorities.

Vertebrate Animals

Not Applicable (No Vertebrate Animals)

Biohazards

Not Applicable (No Biohazards)

Resubmission

- Candidate was highly responsive to past reviews.

Training in the Responsible Conduct of Research

Acceptable

Comments on Format (Required):

- Will complete a 9 hour course at Stanford

Comments on Subject Matter (Required):

- acceptable

Comments on Faculty Participation (Required; not applicable for mid- and senior-career awards):

- Yes

Comments on Duration (Required):

- acceptable

Comments on Frequency (Required):

- acceptable

Select Agents

Not Applicable (No Select Agents

Resource Sharing Plans

Acceptable

Authentication of Key Biological and/or Chemical Resources

Not Applicable (No Relevant Resources)

Budget and Period of Support

Recommend as Requested

CRITIQUE 2

Candidate: 1
Career Development Plan/Career Goals /Plan to Provide Mentoring: 2
Research Plan: 3
Mentor(s), Co-Mentor(s), Consultant(s), Collaborator(s): 1
Environment Commitment to the Candidate: 2

Overall Impact: This revised application proposes training and scientific aims designed to understand the optimal digital approach to self-monitoring in obesity. Training aims are designed to further the candidate's knowledge of MOST and factorial designs, qualitative and mixed methods approaches, and recruitment and retention in clinical trials as well to promote the transition to independence. In addition, the research plan involves a pilot study of 0-3 self-monitoring targets (diet, physical activity and/or body weight) in the context of a weight management program. The mentorship team is strong and there is ample evidence of collaboration and shared models of training. Importantly the candidate is exceptionally strong and well-prepared to continue programmatic work in the area of digital health and obesity treatment.

Overall, the applicant has been responsive to the critiques raised in the prior review. The applicant has improved the focus on recruitment and retention and added training in this area. However, there are some concerns about the utility of the new training plan in recruitment. In addition, there are minor concerns related to the potential synergy of the 3 self-monitoring targets that are not fully addressed in the response. Nonetheless, given the candidate's trajectory to date, the excellent mentoring team and the proposed training in factorial designs and mixed method approaches, it is likely the proposed project will yield significant information to generate further science on a critical issue in obesity treatment.

1. Candidate

Strengths

- The candidate has an excellent training record to date including, awards as a graduate student from SBM and TOS and participation on a competitive T32 fellowship at Stanford Prevention Research Center
- The candidate has a clear history of focused scholarship as evidenced by 14 peer review manuscripts with 6 as first author. Additional manuscripts under review document the candidate's continued productivity.
- Excellent letters of recommendation attest to her career trajectory to date.
- Notably, the candidate has received pilot funding grants, one of which, a project testing recruitment strategy, is relevant to the proposed research aims, was received in the revision period between last review and this application.

Weaknesses

- None noted

2. Career Development Plan/Career Goals & Objectives

[redacted]

Strengths

- The rationale for testing combinations of self-monitoring targets in a clinical trial is excellent and of high clinical and scientific import.
- The use of the MOST design is well rationalized and appropriate to the question.
- The focus on digital tools for self-monitoring is both pragmatic, feasible and scientifically sound.
- Including a qualitative evaluation of barriers//facilitators of engagement in self-monitoring provides unique data that may help further refine the optimal combination of monitoring targets

Weaknesses

- Although the application provides excellent justification for self-monitoring targets selected, there is less attention to the ways in which monitoring more than one target will impact the monitoring of others.
- In addition, although the three targets (diet, steps, and weight) are the most appropriate, there is possibility that the feasibility/acceptability/engagement with monitoring targets may reflect differences in the processes involved in monitoring. That is, monitoring weight typically involves a single assessment/day, while self-monitoring diet is a much more involved construct and can involve multiple entries per day.

4. Mentor(s), Co-Mentor(s), Consultant(s), Collaborator(s)

Strengths

- The mentorship team (Drs. Abby King, Dori Steinberg, Lisa Rosas) are excellent and well-matched to the training plan.
- There is strong evidence of a productive and positive mentoring relationship between the applicant and the primary mentor and clear plans for the team, in conjunction with the primary mentor to promote the candidate's career development.
- The mentorship team and consultants provide the necessary expertise to help the applicant progress through the proposed research trail and training.
- The mentorship team has exceptional scientific resources which will also support the candidate's development.

Weaknesses

- None noted.

5. Environment and Institutional Commitment to the Candidate:

Strengths

- There is ample support in the institution for the research and training.
- The resources at the institution are exceptionally supportive of training.

Weaknesses

- None noted

Study Timeline:

Strengths

- The timeline for the RCT is reasonable and well justified by prior data and experience across the PI and mentorship team.

Weaknesses

- None noted.

Protections for Human Subjects

Acceptable Risks and Adequate Protections

- adequate protections for the study design.

Data and Safety Monitoring Plan (Applicable for Clinical Trials Only):

Acceptable

- low risk study and appropriate monitoring and protections.

Inclusion Plans

- Sex/Gender: Distribution justified scientifically
- Race/Ethnicity: Distribution justified scientifically
- For NIH-Defined Phase III trials, Plans for valid design and analysis: Not applicable
- Inclusion/Exclusion Based on Age: Distribution justified scientifically
- It is not clear how the PI will overrecruit members of specific racial/ethnic identities and how these will affect the embedded test of recruitment strategies.

Vertebrate Animals

Not Applicable (No Vertebrate Animals)

Biohazards

Not Applicable (No Biohazards)

Resubmission

- This resubmitted application has addressed many of the critiques raised in prior review. Notably, the career development plan now provides adequate detail on the transition to independence and plans for specific manuscripts and trainings. In addition, the institutional commitment has been clarified as not conditional on the award and the rate of attrition has been expanded and justification for the proposed retention rates added.

Training in the Responsible Conduct of Research

Acceptable

Comments on Format (Required):

- formal course and informal discussion

Comments on Subject Matter (Required):

- all aspects of RCR mentioned

Comments on Faculty Participation (Required; not applicable for mid- and senior-career awards):

- multiple faculty and mentors

Comments on Duration (Required):

- at least 9 hours

Comments on Frequency (Required):

- acceptable

Select Agents

Not Applicable (No Select Agents)

Resource Sharing Plans

Not Applicable (No Relevant Resources

Authentication of Key Biological and/or Chemical Resources

Not Applicable (No Relevant Resources)

Budget and Period of Support

Recommend as Requested

CRITIQUE 3

Candidate: 2
Career Development Plan/Career Goals /Plan to Provide Mentoring: 2
Research Plan: 3
Mentor(s), Co-Mentor(s), Consultant(s), Collaborator(s): 2
Environment Commitment to the Candidate: 1

Overall Impact: This is a revised K23 award submission from a third-year postdoctoral fellow at the Stanford Prevention Research Center, supported by an NIH T32 fellowship. The applicant got training in clinical psychology and behavioral medicine and published five first-author research articles in the area of remotely delivered digital interventions for weight loss. Applicant's career goal is developing expertise in optimizing digital health interventions for treating obesity and preventing chronic disease. Through this K23 award, the applicant wants to develop expertise in the Multiphase Optimization Strategy (MOST) framework using efficient factorial designs to build multi-component interventions, qualitative and mixed methods research approaches and analytic strategies, and innovative recruitment and retention strategies. The mentoring team is interdisciplinary and highly qualified to support the research outlined in this proposal that involves conducting a 6-month optimization trial that randomizes 176 adults with overweight/obesity to 0-3 self-monitoring components (tracking dietary intake, physical activity, and/or body weight) using a full factorial design. Aim 1 will examine the optimal combination of self-monitoring strategies that maximize 6-month weight loss. Aim 2 will evaluate barriers to and facilitators of engaging in these self-monitoring strategies, which will be assessed via semi-structured qualitative interviews with 40 trial participants. Aim 3 will assess the impact of an interactive video-based recruitment strategy on retention. This is a well-developed proposal with some concerns in the research plan reducing enthusiasm. The proposed factorial design will result in 22 participants in each group (i.e., 8 combinations) leading to limited power in evaluating the impact of individual self-monitoring components and their interactions. The limited number of subjects in each group will not

allow robust analysis after taking into consideration of other confounding factors such as age, gender, other comorbid conditions (e.g., diabetes, hypertension, neurological conditions). How the subjects in each group will be matched to avoid the impact of confounding factors on the results is not clear in the proposal? I am not sure if the sample size of 176 with an 8-way factorial design will be able to provide any insight into key factors associated with weight loss, therefore a larger sample size or limited factorial (i.e., 3 combination) will be required for gaining any meaningful insights. For all factorial analysis, the applicant should be blinded to the group information, how this will be handled in this digital data gathering schema is not clear from analytical section of the project.

Similarly, in Aim 2 evaluation of barriers to and facilitators of engaging in these self-monitoring strategies is also severely underpowered and might fail to provide any insight therefore it should be expanded to all subjects in the trial.

1. Candidate

Strengths

- None noted

Weaknesses

- None noted

2. Career Development Plan/Career Goals & Objectives:

Strengths

- None noted

Weaknesses

- None noted

3. Research Plan:

Strengths

- None noted

Weaknesses

- None noted

4. Mentor(s), Co-Mentor(s), Consultant(s), Collaborator(s):

Strengths

- None noted

Weaknesses

- None noted

5. Environment and Institutional Commitment to the Candidate:

Strengths

- Superb

Weaknesses

- None noted

Study Timeline:

Strengths

- Adequate

Weaknesses

- None noted

Protections for Human Subjects

Acceptable

Data and Safety Monitoring Plan (Applicable for Clinical Trials Only)

Acceptable:

Inclusion Plans

- Sex/Gender: Distribution justified scientifically
- Race/Ethnicity: Distribution justified scientifically
- For NIH-Defined Phase III trials, Plans for valid design and analysis: Not applicable
- Inclusion/Exclusion Based on Age: Distribution justified scientifically

Vertebrate Animals

- Not Applicable

Biohazards

- None noted

Resubmission

Partially Responsive to previous review

Training in the Responsible Conduct of Research

Acceptable

Comments on Format (Required):

Adequate

Comments on Subject Matter (Required):

Adequate

Comments on Faculty Participation (Required; not applicable for mid- and senior-career awards):

Adequate

Adequate

Comments on Duration (Required):

Adequate

Comments on Frequency (Required):

Adequate

Select Agents

Not Applicable

Resource Sharing Plans

Acceptable

Authentication of Key Biological and/or Chemical Resources

Not Applicable

Budget and Period of Support

Recommended as Requested

THE FOLLOWING SECTIONS WERE PREPARED BY THE SCIENTIFIC REVIEW OFFICER TO SUMMARIZE THE OUTCOME OF DISCUSSIONS OF THE REVIEW COMMITTEE, OR REVIEWERS' WRITTEN CRITIQUES, ON THE FOLLOWING ISSUES:

PROTECTION OF HUMAN SUBJECTS: ACCEPTABLE
The reviewers recommended that an independent safety officer would be beneficial for the DSMP.

INCLUSION OF WOMEN PLAN: ACCEPTABLE

INCLUSION OF MINORITIES PLAN: ACCEPTABLE

INCLUSION ACROSS THE LIFESPAN: ACCEPTABLE

SCIENTIFIC REVIEW OFFICER'S NOTES:
The plans outlined in the application to obtain training in the responsible conduct of research are adequate to satisfy this requirement.

The resource sharing plan is adequate.

The authentication plan for key biologicals and/or chemical resources is not applicable.

COMMITTEE BUDGET RECOMMENDATIONS: The budget was recommended as requested.

Recommended direct cost levels are estimated and are subject to further adjustment based on the Institute's standard budget calculation practices.

Footnotes for 1 K23 DK129805-01A1; PI Name: Patel, Michele Lanpher

NIH has modified its policy regarding the receipt of resubmissions (amended applications).See Guide Notice NOT-OD-18-197 at https://grants.nih.gov/grants/guide/notice-files/NOT-OD-18-197.html. The impact/priority score is calculated after discussion of an application by averaging the overall scores (1-9) given by all voting reviewers on the committee and multiplying by 10. The criterion scores are submitted prior to the meeting by the individual reviewers assigned to an application, and are not discussed specifically at the review meeting or calculated into the overall impact score. Some applications also receive a percentile ranking. For details on the review process, see http://grants.nih.gov/grants/peer_review_process.htm#scoring.
